# Ammonium tetrathiomolybdate following ischemia/reperfusion injury: Chemistry, pharmacology, and impact of a new class of sulfide donor in preclinical injury models

**DOI:** 10.1371/journal.pmed.1002310

**Published:** 2017-07-05

**Authors:** Alex Dyson, Felipe Dal-Pizzol, Giovanni Sabbatini, Anna B. Lach, Federica Galfo, Juliano dos Santos Cardoso, Bruna Pescador Mendonça, Iain Hargreaves, Bernardo Bollen Pinto, Daniel I. Bromage, John F. Martin, Kevin P. Moore, Martin Feelisch, Mervyn Singer

**Affiliations:** 1 Bloomsbury Institute for Intensive Care Medicine, Division of Medicine, University College London, London, United Kingdom; 2 Magnus Oxygen, London, United Kingdom; 3 Laboratory of Experimental Pathophysiology, University of Southern Santa Catarina, Criciúma, Brazil; 4 Department of Clinical and Experimental Medicine, University of Messina, Messina, Italy; 5 Department of Molecular Neuroscience, Institute of Neurology, University College London, London, United Kingdom; 6 Hatter Cardiovascular Institute, University College London, London, United Kingdom; 7 Institute for Liver and Digestive Health, University College London, London, United Kingdom; 8 Clinical and Experimental Sciences, Faculty of Medicine, Southampton General Hospital and Institute for Life Sciences, University of Southampton, Southampton, United Kingdom; Barts and the London School of Medicine & Dentistry Queen Mary University of London, UNITED KINGDOM

## Abstract

**Background:**

Early revascularization of ischemic organs is key to improving outcomes, yet consequent reperfusion injury may be harmful. Reperfusion injury is largely attributed to excess mitochondrial production of reactive oxygen species (ROS). Sulfide inhibits mitochondria and reduces ROS production. Ammonium tetrathiomolybdate (ATTM), a copper chelator, releases sulfide in a controlled and novel manner, and may offer potential therapeutic utility.

**Methods and findings:**

In vitro, ATTM releases sulfide in a time-, pH-, temperature-, and thiol-dependent manner. Controlled sulfide release from ATTM reduces metabolism (measured as oxygen consumption) both in vivo in awake rats and ex vivo in skeletal muscle tissue, with a superior safety profile compared to standard sulfide generators. Given intravenously at reperfusion/resuscitation to rats, ATTM significantly reduced infarct size following either myocardial or cerebral ischemia, and conferred survival benefit following severe hemorrhage. Mechanistic studies (in vitro anoxia/reoxygenation) demonstrated a mitochondrial site of action (decreased MitoSOX fluorescence), where the majority of damaging ROS is produced.

**Conclusions:**

The inorganic thiometallate ATTM represents a new class of sulfide-releasing drugs. Our findings provide impetus for further investigation of this compound as a novel adjunct therapy for reperfusion injury.

## Abstract 2

In preclinical rat injury models Alex Dyson and colleagues investigate the potential of ammonium tetrathiomolybdate to protect organs from injury after restoration of blood flow following ischemia.

## Author summary

**Why was this study done?:**

- Reperfusion injury results from restoration of blood flow to ischemic organs, e.g., following myocardial or cerebral revascularization or resuscitation from hemorrhage. Reperfusion injury may be as severe as the initial ischemic event, yet there is no currently approved treatment.
- Sulfide-releasing drugs are known to be effective against reperfusion injury in animal models, but none to date have shown efficacy in patients, and safety concerns have been raised.

**What did the researchers do and find?:**

- We found that ammonium tetrathiomolybdate (ATTM) releases sulfide in a controlled manner and potentially fits the desired efficacy and safety profile. The chemical nature of sulfide release from ATTM differs from that of other sulfide-releasing drug classes.
- In our animal models, ATTM decreased oxygen metabolism and protected against reperfusion injury and organ damage following heart attack, stroke, and hemorrhage, with significant improvements in outcome, and a good safety profile.

**What do these findings mean?:**

- We have discovered a novel class of sulfide-releasing drugs. In our animal models, ATTM protected body organs from damage induced by reperfusion of ischemic tissues.
- ATTM could be potentially used in patients as a short-term protective therapy while blocked blood vessels feeding major organs such as the brain and heart are being opened up, or during resuscitation from severe bleeding. Effectiveness and safety (short- and long-term) need to be explored in humans.
- If ATTM can be successfully developed, it could significantly reduce long-term organ damage after heart attack, stroke, hemorrhage, and other ischemia/reperfusion conditions.

## Introduction

Ischemia/reperfusion (I/R) injury is well recognized following multiple therapeutic interventions such as revascularization following myocardial ischemia or cerebrovascular thrombosis, resuscitation following hemorrhage, and organ transplantation. Following injurious reduction of blood flow to tissues, further damage is induced by reperfusion, necessarily caused by restoration of an adequate oxygen supply. Reperfusion injury is largely attributed to excess production of reactive oxygen species (ROS) by mitochondria [1].

The development of highly effective reperfusion therapies such as percutaneous coronary intervention and thrombolysis has revolutionized acute management. However, residual morbidity from both irreversible ischemia (from delayed intervention) and reperfusion injury (from the intervention itself) still represents a considerable social and economic healthcare burden, leading to chronic organ failure and a shortened lifespan. Indeed, of noncommunicable diseases, the most recent Global Burden of Disease Study cites cardiovascular disease as the leading cause of mortality worldwide [2]. Multiple strategies to prevent or minimize reperfusion injury have to date proved unsuccessful in clinical trials [3,4].

A landmark paper in 2005 showed that inhalation of H_2_S by mice induced a profound, reversible hypometabolic state of “suspended animation” [5]. This effect is mediated by inhibition of cytochrome C oxidase, the terminal complex of the mitochondrial respiratory chain [6]. H_2_S is moderately water-soluble, partitioning between aqueous and gas/lipid compartments; as a weak acid, it deprotonates following dissolution to form a hydrosulfide anion (HS^−^), with negligible concentrations of S^2−^ at physiological pH [7]. Unless stated otherwise, all three forms herein are referred to as “sulfide.” Applied either in the form of gaseous H_2_S or as an intravenous (IV) sodium salt (Na_2_S), sulfide was shown to protect against lethal hemorrhage [8] and hypoxia [9] in small rodent models. However, attempts to induce hypometabolism and improve outcomes in larger mammals following severe cardiorespiratory insults yielded variable results [10,11]. A clinical trial of Na_2_S in coronary artery bypass surgery was stopped prematurely in 2010 [12] for reasons still not published. A burgeoning literature on novel sulfide-releasing drugs reports encouraging results in experimental studies of I/R injury [13], though none of these newer products have yet been tested clinically.

Thiomolybdates were first synthesized nearly two centuries ago [14]. The ammonium salt, ammonium tetrathiomolybdate (ATTM) ([NH_4_]_2_MoS_4_), has been used off-label for decades as an oral copper chelator to treat Wilson disease in humans [15] and shows utility against copper toxicity in sheep [16]. In three patients with Wilson disease, IV dosing of ATTM was reported to have a copper-lowering effect with no reported safety concerns [17]. In animal models, ATTM has proven beneficial across myriad experimental pathologies including fibrotic, autoimmune, and malignant diseases; copper-handling ability, with subsequent anti-inflammatory and anti-angiogenic effects, is presumed responsible for its efficacy [18]. Upon dissolving ATTM into aqueous solution, we noted the pungent rotten egg odor characteristic of sulfide [19], a finding not formally documented in the medical literature. Our characterization of ATTM revealed that it belongs to a new class of inorganic sulfide-releasing molecules, the thiometallates, in which one or more sulfur atoms are covalently bound to a transition metal. We hypothesized that ATTM could (1) reduce metabolism through graded inhibition of oxidative phosphorylation, (2) confer protection in both whole animal and organ-specific models of I/R injury, and (3) offer a superior safety profile to a benchmark sulfide generator.

## Methods

### Ethics, animals, and husbandry

Male Wistar rats (approximately 300 g body weight) were used in all in vivo studies. Use of this species allows greater blood sampling than is possible with mice. Importantly, mice markedly and rapidly reduce their metabolism in response to a severe systemic insult, whereas rats more closely mimic human responses [20]. Use of mice would likely confound evaluation of a metabolism-modifying agent. All experiments (except brain I/R studies) were performed in the UK according to local ethics committee (University College London) and UK Home Office guidelines under the Animals (Scientific Procedures) Act 1986. Brain I/R studies (performed in Brazil) were carried out in accordance with Brazilian National Council for the Control of Animal Experimentation (CONCEA) guidelines. Animals used for UK- and Brazil-based studies were purchased from Charles River Laboratories (Margate, UK) or bred in-house at the University of Southern Santa Catarina (Criciúma, Brazil), respectively. All animals were healthy and certified pathogen-free, and housed in cages of four individuals on a 12-h light/dark cycle, with food and water ad libitum prior to experimentation. Standard cages and bedding were used. Additional tissue paper was provided for comfort, and cardboard tubes for cage enrichment. All experiments were started at 9 a.m. local time. All animals were anesthetized with isoflurane (Abbott, Maidenhead, UK). Where mechanical ventilation was used, the anesthetic was switched to intraperitoneal sodium pentobarbitone (Pentoject; Animalcare, York, UK) just prior to intubation. Perioperative analgesia was provided by buprenorphine, 0.05 mg/kg subcutaneously (Reckitt Benckiser, Slough, UK), or dipyrone, 80 mg/kg subcutaneously (Laboratório Teuto Brasileiro, Anápolis, Brazil). Euthanasia at experiment end was performed by IV sodium pentobarbitone.

### In vitro sulfide release

Free sulfide (H_2_S gas) liberated from ATTM or NaHS (sodium hydrosulfide; Alfa Aesar, Heysham, UK) was measured following dissolution of the compounds in phosphate-buffered saline at room temperature; the H_2_S gas was captured in the headspace of airtight (further sealed with Parafilm; Bemis, Neenah, WI) 50-ml Falcon tubes (Corning Science Mexico, Reynosa, Mexico). Aliquots of the ATTM stock were rapidly diluted 1:10 with phosphate-buffered saline that was pH- or temperature-adjusted or contained thiols (L-cysteine or reduced glutathione [GSH], both 5 mM final concentration), as required (*n* = 3–6/group). The liquid and gas (headspace) phases comprised 5 and 45 ml, respectively. Following incubation, 5 ml of headspace gas was sampled and passed through a commercially available H_2_S detector (Z900XP; Environmental Sensors, Boca Raton, FL); peak H_2_S gas concentrations were recorded. We used ATTM purchased from a single supplier (Sigma-Aldrich, Gillingham, UK) on all but one occasion. Since the compound purchased elsewhere (Strem Chemicals, Newburyport, MA) was chemically dissimilar (ultraviolet—visible spectroscopy and sulfide release), we used the Sigma-Aldrich compound for all experiments. Unless stated otherwise, all other chemicals/reagents were purchased from Sigma-Aldrich.

In separate experiments, total sulfide concentrations from ATTM (*n* = 6) and NaHS (*n* = 5) at different pH levels (diluted as above, with a liquid:gas phase ratio of 0.67:0.33) were obtained via incubation, derivatization, then measurement using a high-performance liquid chromatography (HPLC)–based monobromobimane (MBB) assay, as previously described [21,22], modified and elaborated upon as described in S1 Text. We used 0.55 mM ATTM and 2.2 mM NaHS to yield comparable quantities of total sulfur.

### In vivo sulfide detection

Anesthetized animals underwent carotid arterial and jugular venous cannulation for blood sampling and drug administration, and a tracheostomy to secure the airway; a more detailed description is provided in S1 Text. After 30 min stabilization, either ATTM (0.25 mg/kg, *n* = 4) or NaHS (1 mg/kg, *n* = 3) was dissolved in 0.9% saline and (within 2 min) administered as an IV bolus (1 ml/kg) over 5 s. Plasma was obtained to measure sulfide concentrations at frequent intervals from 15 s to 120 min post-administration. In separate studies, ATTM (10 or 20 mg/kg/h, *n* = 4/group) was continually infused intravenously, with samples taken from 5 to 180 min after infusion onset.

### Ex vivo metabolic study

Soleus muscle tissue was obtained from anesthetized animals and dissected, and the resulting fibers permeabilized with saponin. After 20 min incubation, saponin and its metabolites were removed by washing the fibers three times in ice-cold respiratory medium (see S1 Text for further detail). Oxygen consumption (VO_2_) was assessed in small bundles of tissue using a Clark-type oxygen electrode (Rank Brothers, Bottisham, UK); this was connected to a sealed chamber and maintained at 37°C. VO_2_ was determined as the fall in oxygen concentration within the chamber over time. Substrates for mitochondrial respiratory chain complexes I and II were added to the medium, which was subsequently oxygenated to 250 μM O_2_ using 100% oxygen gas. Two series of experiments were performed. In the first, VO_2_ was assessed following vehicle (*n* = 8) or increasing concentrations of ATTM (2–32 mM total sulfur, *n* = 8), NaHS (0.5–4 mM, *n* = 3), or combined non-sulfide-containing copper chelators (neocuproine and cuprizone, both 1–100 μM). In the second, tissues respired to hypoxia following addition of either vehicle (*n* = 12), ATTM (2 mM total sulfur, *n* = 9), NaHS (0.5 mM, *n* = 4), or copper chelators (100 μM, *n* = 7), added at 200 μM O_2_. Oxygen consumption was determined from 175 to 75 μM O_2_ at 25 μM intervals and compared to baseline values recorded prior to the addition of drugs.

### In vivo metabolic study

Anesthetized animals had arterial and venous lines tunneled subcutaneously to the nape of the neck. The lines were then attached to a swivel tether system (Linton Instrumentation, Diss, UK), and the animals then placed into metabolic chambers (Oxymax system; Columbus Instruments, Columbus, OH), where they were allowed to recover. These chambers enable continuous measurement of whole body O_2_ consumption. Animals were acclimatized for 24 h and frequently assessed using an in-house clinical scoring system before inclusion into two separate studies (*n* = 4/group). Short-term changes in O_2_ consumption were assessed following increasing, hourly IV bolus doses of ATTM (2–20 mg/kg). Controls received equivalent volumes of saline (2 ml/kg per bolus). Longer-term effects on oxygen consumption were assessed following continuous IV infusions of ATTM (10 mg/kg/h for 24 h) or an equivalent volume of saline (10 ml/kg/h). At the end of each long-term metabolic experiment, animals were re-anesthetized, core temperature was measured rectally, and heart rate determined using echocardiography (see S1 Text).

### Pharmacokinetic/pharmacodynamic and safety studies

Mechanically ventilated animals were anesthetized with isoflurane during instrumentation and with sodium pentobarbitone thereafter. After a stabilization period, they received increasing IV bolus doses of ATTM (1–100 mg/kg, *n* = 3), the bis-choline salt of ATTM (ATN-224, 1–100 mg/kg, *n* = 3), or NaHS (0.01–1 mg/kg, *n* = 4). Ventilator (PhysioSuite; Kent Scientific, Torrington, CT) settings were as follows: tidal volume, 10 ml/kg; respiratory rate, 80/min; and positive end-expiratory pressure, 3 cm H_2_O. H_2_S in exhaled breath was measured by connecting the ventilator exhaust to the (above) H_2_S detector. Measurements were collected at baseline, then as follows after each dose: blood pressure and exhaled H_2_S (peak change or level) within 30 s, myocardial function (by echocardiography [see S1 Text]) within 1 min, blood sampling to determine plasma concentration at 2 min, and arterial blood gas analysis (to measure partial pressure of O_2_ [PaO_2_] and CO_2_ [PCO_2_], glucose, lactate, and acid/base changes) at 27 min; doses were escalated every 30 min. For ATTM and ATN-224, the absorbance (at 468 nm) of plasma samples was assessed using a microplate reader and BioTek (Gen5) software (Synergy 2, North Star Scientific, Sandy, UK). Plasma concentrations were derived by comparison against standard curves. In separate studies assessing safety, spontaneously breathing, instrumented rats received either ATTM (10 mg/kg IV bolus, then 20 mg/kg/h, *n* = 11) or 0.1 M hydrochloric acid (10 ml/kg/h, *n* = 5) for 5 h. A subset of ATTM-treated animals received 70% inspired oxygen from 3 h onwards (*n* = 7). Vehicle-treated animals (*n* = 10) received equivalent volumes of 0.9% saline. Blood pressure, echocardiography, arterial blood gas analysis, and vastus lateralis tissue oxygen tension (measured using an indwelling fiber-optic sensor; Oxford Optronix, Abingdon, UK) were determined at baseline, then hourly. Whole blood was removed (at baseline, 3 h, and 5 h) and stored (−80°C) for subsequent measurement of sulfhemoglobin.

### Sulfhemoglobin

In vivo samples were defrosted and immediately diluted (1:10) in human erythrocyte lysing solution [23]. After 5 min, samples were centrifuged, and sulfhemoglobin measured by absorbance of the supernatant at 620 nm, using a microplate reader, as above. To express the sulfhemoglobin level as a percentage, oxyhemoglobin level was measured at 577 nm in baseline samples to obtain total hemoglobin, and adjusted for protein content based on blood gas machine-derived values at later time points. In separate experiments, concentration response curves were constructed using naïve rat blood, spiked and incubated (at 37°C for 60 min) with either ATTM or NaHS (0.3–100 mM total sulfur, *n* = 3/group). Sulfhemoglobin was subsequently measured as above.

### Myocardial and cerebral ischemia/reperfusion

For the myocardial model, anesthetized (with isoflurane during instrumentation and sodium pentobarbitone thereafter), mechanically ventilated rats (settings as above) received IV fluids (2.5% glucose in 0.9% saline infused throughout; 10 mg/kg/h). Blunt thoracotomy of the left fourth intercostal space was performed to access the thoracic cavity. The pericardium was removed, and the left anterior descending (LAD) coronary artery ligated with 6–0 Mersilk suture (Ethicon, Edinburgh, UK) 8 mm from the apex for 30 min. Reperfusion was initiated by untying the LAD suture. The chest wall was then closed. At experiment end (4 h post-reperfusion), blood was removed for biomarker analysis, the chest reopened, the pulmonary artery ruptured, and the heart retrogradely perfused via the carotid artery with 10 ml of 0.9% saline. The LAD suture was then closed, and the heart perfused in vivo with Evans blue dye (1 ml of a 0.5% solution). Hearts were excised and stored for later batch analysis of area at risk and infarct size.

For brain I/R studies, animals were anesthetized and instrumented, and ischemia initiated by bilateral carotid artery occlusion for 25 min. Animals then recovered, receiving analgesia immediately and 12 h after surgery (dipyrone, 80 mg/kg subcutaneously). At experiment end (24 h post-reperfusion), animals were re-anesthetized, blood was removed for biomarker analysis (by cardiac puncture), and brains were removed for assessment of infarct size.

For both organ-specific models, animals were randomized to receive vehicle (0.9% saline, *n* = 6/group) or ATTM treatment (10 mg/kg IV bolus, *n* = 6/group) immediately prior to reperfusion, thus mimicking a clinical scenario. ATTM-treated animals received a further IV infusion (10 mg/kg/h) for 60 min, equaling a total dose of 20 mg/kg. Control animals received equivalent volumes of 0.9% saline.

Four heart (distal to the LAD suture) and brain slices were incubated with 0.1% tetrazolium chloride for 20 min, then fixed with 10% formalin for 30 min. Open access software (Image J, 1.49e; National Institutes of Health, Bethesda, MD) was used to quantify the area at risk (not perfused with Evans blue) and viable tissue (stained with tetrazolium chloride). For the heart model, we prospectively constrained the area at risk as a proportion of the left ventricular area studied to fall within 1.5 standard deviations of the median. This equated to values between 25% and 75%, with outliers (10% of cases) being excluded from further analysis. B-type natriuretic peptide (BNP; for heart) and S100 calcium binding protein β (S100β; for brain) were assayed using commercially available kits (Life Diagnostics, Stoke-on-Trent, UK); assays were performed in duplicate and according to the manufacturer’s instructions.

### Global ischemia/reperfusion

Anesthetized, spontaneously breathing animals were subjected to global ischemia by removal of 50% of estimated blood volume (35 ml/kg) over 15 min, then were monitored for a further 90 min before randomization to receive ATTM (10 mg/kg IV, *n* = 16) or an equivalent volume of saline (*n* = 16). If the mean arterial blood pressure fell below 40 mm Hg during the ischemia phase, animals were administered 1 ml/kg of 0.9% saline IV. Reperfusion was commenced by re-infusing autologous shed blood over 15 min. In ATTM-treated animals, the blood was supplemented with a further 2.5 mg/kg, equaling a total dose of 12.5 mg/kg. The primary endpoint for this study was 6 h mortality, with the animals kept under anesthesia throughout. Animals were monitored with measurements (as above) performed at baseline, before reperfusion, and hourly after reperfusion. Blood (replaced by twice volume 0.9% saline) was removed 2 h post-reperfusion for measurement of interleukin-6 (IL-6) and plasma markers of oxidative stress and redox status (protein carbonyls, oxidized glutathione [GSSG], and GSH). IL-6 (Millipore, Billerica, MA) and protein carbonyls (OxiSelect Protein Carbonyl Spectrophotometric Assay; Cell Biolabs, San Diego, CA) were measured using standard bioassay kits and assayed in duplicate; assays were performed according to the manufacturer’s instructions. GSH and total glutathione were measured in whole blood using HPLC (Jasco, Easton, MD) with fluorescence detection—excitation and emission at 385 and 515 nm, respectively—with HPLC columns (C18) and glutathione detection kits from Chromsystems Instruments & Chemicals (Gräfelfing, Germany).

### In vitro anoxia/reoxygenation

Cultured H9C2 (cardiomyoblast) cells were plated (onto six-well Corning Costar cell culture plates) at a density of 60 × 10^3^/well. Culture medium was replaced with serum-free Dulbecco’s Modified Eagle Medium (Thermo Fisher Scientific, Loughborough, UK) 24 h later, and adherent cells placed into an anoxic chamber (95% N_2_ and 5% CO_2_) for a further 24 h. Reoxygenation was initiated by substitution with fresh (“normoxic”) culture medium supplemented with ATTM (0.055–5.5 mM, *n* = 3/group). Viability was assessed 2 h post-reoxygenation using an Annexin V—propidium iodide—based assay. Briefly, cells were washed, resuspended in 100 μl of allophycocyanin—Annexin V (BD Biosciences, Oxford, UK) binding buffer (150 mM NaCl, 10 mM HEPES [pH 7.4], 10 mM CaCl_2_), and stained for 15 min at room temperature in dark conditions. To assess ROS production, cells were loaded with MitoSOX Red mitochondrial superoxide indicator (5 μM; Thermo Fisher Scientific, Loughborough, UK) for 30 min [24]. Data were acquired and analyzed by flow cytometry (FACSCalibur; BD CellQuest, BD Biosciences, Oxford, UK) and FlowJo (v. IX; FlowJo, Ashland, OR), respectively.

### Study design, data, and statistics

In vivo I/R and metabolic studies were performed using 1×1 and 2×2 study designs, respectively. For in vivo studies with >2 treatment groups, animals were randomly allocated on the day of experimentation. Experiments could not be performed in a blinded fashion due to the distinctive coloration and sulfurous odor of ATTM. However, all biochemistry (biomarker measurement) and histology (infarct size determination) studies were undertaken by investigators unaware of the treatment allocation. Data are presented as mean ± standard error or median, quartiles, and range. Pharmacokinetic data were analyzed using a two-phase decay curve and least squares fitting method. Survival times were analyzed using a log-rank test. Parametric data were analyzed using repeated measures one- or two-way ANOVA followed by Bonferroni’s post hoc testing, as appropriate. Nonparametric data were analyzed using the Mann—Whitney U test. All statistical analyses were two-tailed and were performed using Prism 7.0.1 software (GraphPad Software, San Diego, CA). This software considers the number of comparisons and automatically adjusts the probability value following multiple comparisons. The number of comparisons varied with the study design. Where a number of treatment groups (*n*) are solely compared to the control arm, the α-level is defined, as per usual convention, as α/*n*. Where comparison is sought within groups, *n* reflects the total number of comparisons. The *p*-values reported herein following multiple comparisons are “multiplicity-adjusted” rather than “exact” *p*-values. After adjustment (as required), *p*-values < 0.05 were considered statistically significant.

## Results

### Sulfide release studies

**Fig 1:**
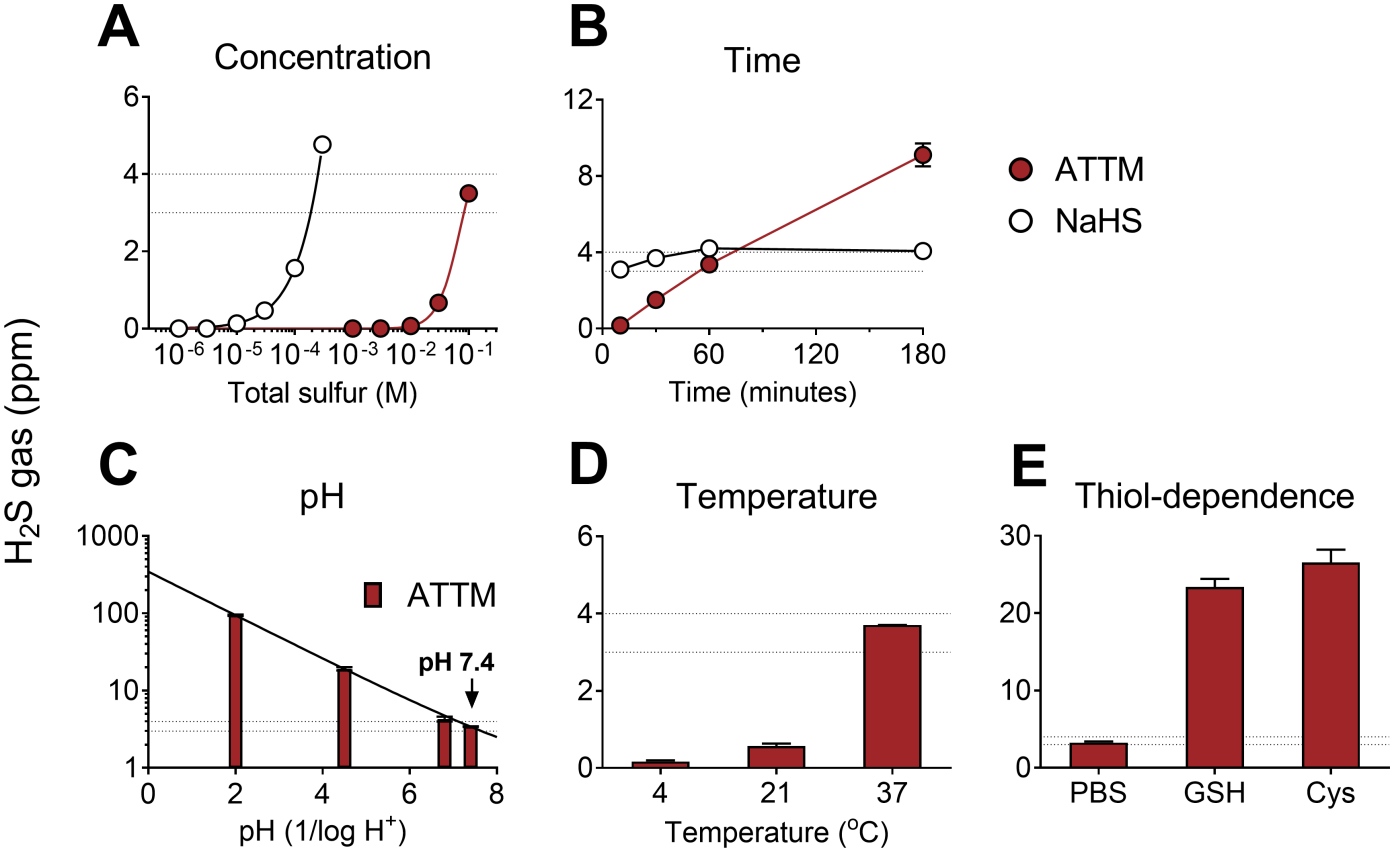
In vitro release of gaseous H_2_S from ATTM and NaHS under different environmental conditions. Comparison of ammonium tetrathiomolybdate (ATTM) and NaHS with changes in (A) concentration and (B) time. In (A), the molarity of each compound was adjusted for equal total sulfur content; drugs were incubated for 1 h at physiological pH (7.4) and temperature (37°C). In (B), fixed concentrations were used: ATTM 100 mM (total sulfur) and NaHS 0.3 mM. The effects of pH, temperature, and the presence of thiols on H_2_S gas released from ATTM are shown in (C–E). Here, fixed concentrations (100 mM total sulfur) and incubation time (1 h) were employed. Peak H_2_S concentrations are displayed in parts per million (ppm). The thiols used were reduced glutathione (GSH; 5 mM) and L-cysteine (Cys; 5 mM). The dotted lines reflect typical H_2_S gas levels (3–4 ppm) obtained from ATTM (100 mM total sulfur) following 1 h incubation at normal physiological pH and temperature. *n* = 3–6 per group.

We first assessed sulfide release from ATTM or NaHS in vitro by measuring H_2_S concentrations in the headspace of airtight vials containing buffered aqueous solutions. When dissolved and incubated at physiological pH and temperature for 1 h, the amount of H_2_S generated was, as expected, dependent on the concentration for both compounds. However, at this time point, approximately 300 times the amount of ATTM (adjusted for total sulfur content) was required to achieve H_2_S levels comparable to those observed with NaHS (Fig 1A). At a fixed concentration, H_2_S release from ATTM was dependent on time, pH, and temperature, with more acidic and warmer conditions favoring sulfide release (Fig 1B–1D). ATTM released sulfide slowly, in a linear manner over time, whereas H_2_S levels from NaHS were rapidly generated and, consequently, similar at all time points (Fig 1B). Co-incubation with thiols (either L-cysteine or GSH) resulted in a 6-fold increase in H_2_S release from ATTM (Fig 1E).

To confirm that sulfide release from ATTM was not related to impurities of a particular batch, we tested five different vials from the same commercial supplier (Sigma-Aldrich) obtained over a 3-y period. With the exception of an additional batch where the compound was purchased elsewhere (see Methods), gaseous H_2_S production was similar when incubated for 1 h under physiological conditions (3–4 ppm, *n* = 3/group; S1 Fig).

Sulfide release from ATTM was further confirmed both in vitro and in vivo using the gold-standard MBB assay [21]. We modified this assay to include L-cysteine to facilitate sulfide detection by removing excess MBB before chromatographic separation. This induces thiol-dependent release of sulfide from ATTM; between pH 4.5 and 10 in vitro, ATTM releases approximately 80% of its bound sulfur as sulfide under these conditions (S2A Fig). While sulfide released from ATTM is detectable using an alternative methodology, our modified assay reflects total, rather than free, sulfide. NaHS, the sulfide generator, was not affected by changes in pH, with 90% of total sulfide recovered in vitro (between pH 2 and 10) using the MBB assay.

ATTM elevated MBB-derived plasma sulfide levels when given as either an IV bolus (0.25 mg/kg; S3A Fig) or by continuous infusion (10 and 20 mg/kg/h; S3B Fig). The pharmacokinetic profile of ATTM following IV bolus administration showed that disappearance from the bloodstream was biphasic, yielding distribution (fast) and elimination (slow) half-lives of 1 and 20 min, respectively (S3D Fig). By contrast, the distribution half-life of NaHS was three times faster than that of ATTM; since plasma sulfide levels normalized within 1 min post-administration (S3C and S3D Fig), the elimination half-life was not determined. Notably, no adverse events were recorded using these doses of ATTM. Bolus IV doses of NaHS in excess of 1 mg/kg resulted in mortality.

### Modulation of metabolism

**Fig 2:**
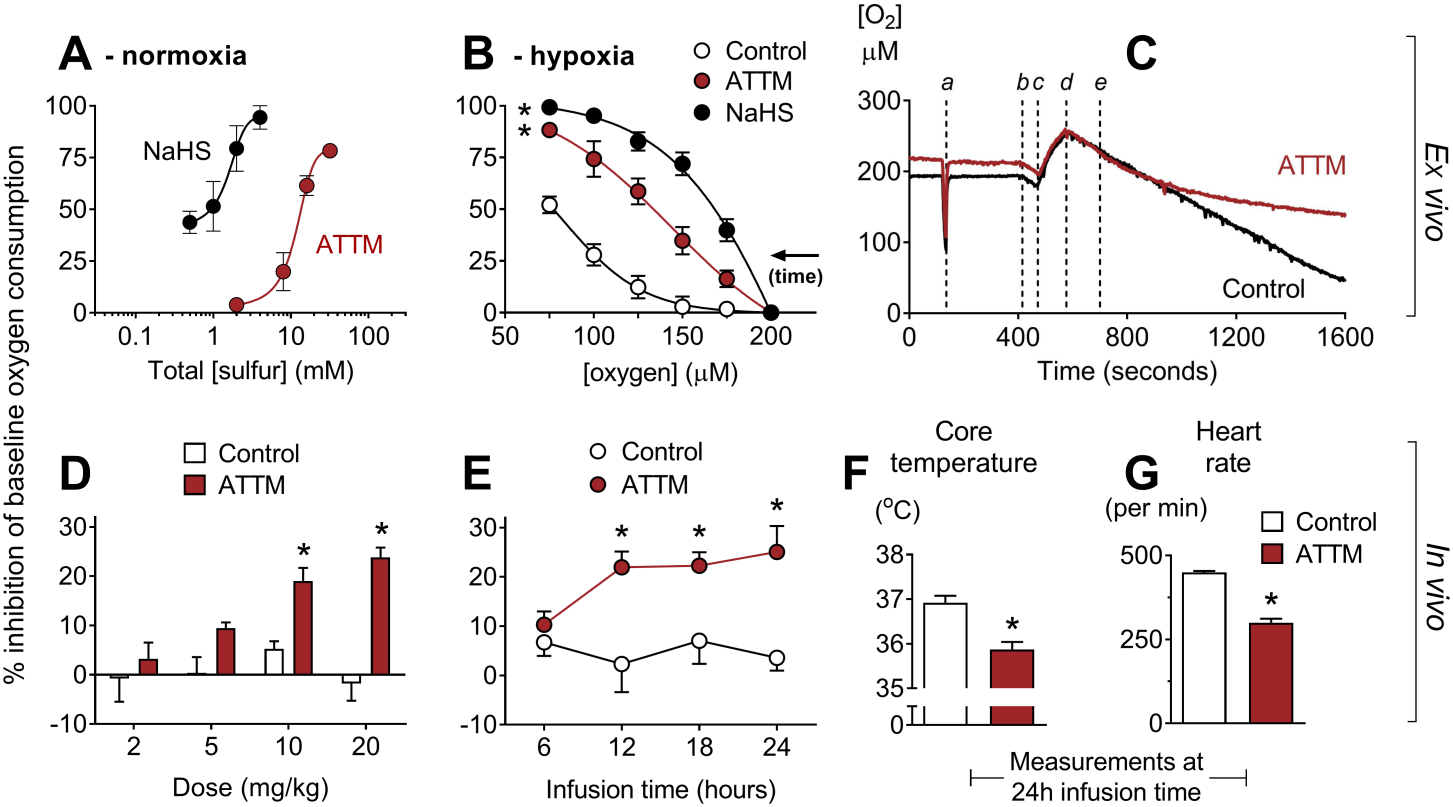
Inhibition of oxygen consumption by sulfide-containing drugs. (A) Ex vivo concentration response curves for ammonium tetrathiomolybdate (ATTM) and NaHS in relative normoxia. Experiments were performed at 150–250 μM O_2_. In (B), tissues respired to hypoxia. Vehicle, ATTM (0.5 mM, corresponding to 2 mM total sulfur), or NaHS (0.5 mM) was added at 200 μM O_2_. Note that oxygen consumption in vehicle-treated tissues also decreases at lower [O_2_] (supply dependence) when tissues respire towards hypoxia. (C) shows a representative trace of tissues respiring to hypoxia, with the timing of the following events indicated: *a*, sensitivity test; *b*, addition of tissue to the chamber; *c*, oxygenation; *d*, baseline measurements; *e*, addition of ATTM or vehicle (control). (D) and (E) show the effects of ATTM in vivo following increasing, hourly IV bolus doses or a continuous infusion (10 mg/kg/h), respectively. (F) shows core temperature and (G) shows echocardiography-derived heart rate at the end (24 h) of continuous infusion. Panels A, B, D, and E show percentage inhibition compared to baseline values, before the addition of drugs. *p < 0.05 versus control using a two-way repeated measures ANOVA (plus Bonferroni’s test in D and E) or unpaired *t*-test (in F and G). *n* = 3–12 for ex vivo experiments, and *n* = 4 per group for in vivo studies.

We assessed the impact of ATTM on metabolism using a range of ex vivo and in vivo assays. Both NaHS and ATTM inhibited oxygen consumption ex vivo in permeabilized rat soleus muscle, with an IC_50_ (concentration causing 50% inhibition) of 1.6 and 11.6 mM, respectively (Fig 2A). As oxygen consumption is dependent on [O_2_] < 150 μM in this model, these experiments were performed in relative “normoxia,” between 150 and 250 μM O_2_. In separate experiments, we applied a single concentration and allowed tissues to respire to hypoxia. ATTM (2 mM total sulfur) and NaHS (0.5 mM) significantly (*p <* 0.05) inhibited oxygen consumption (versus vehicle; Fig 2B). Notably, the concentration of ATTM that could inhibit oxygen consumption in “hypoxic” tissues (Fig 2B; example trace, Fig 2C) had no effect under normoxic conditions (Fig 2A).

Combined treatment with the non-sulfide-containing copper chelators neocuproine (Cu^1+^) and cuprizone (Cu^2+^) had no effect in either model (S4A and S4B Fig; *p* = 0.30 and 0.48, respectively).

To assess oxygen consumption in vivo, we placed awake, tethered, unrestrained rats into metabolic cages. ATTM, given either as increasing IV bolus doses (Fig 2D) or as a continuous infusion (Fig 2E), inhibited the whole body oxygen consumption of freely moving animals by 25% (*p <* 0.05). In rats that had received a continuous infusion for 24 h, we found that, compared to vehicle-treated controls, core temperature decreased (Fig 2F; *p <* 0.05) and heart rate (measured by echocardiography) fell by one-third (Fig 2G; *p <* 0.05).

### Pharmacokinetics and pharmacodynamics

**Fig 3:**
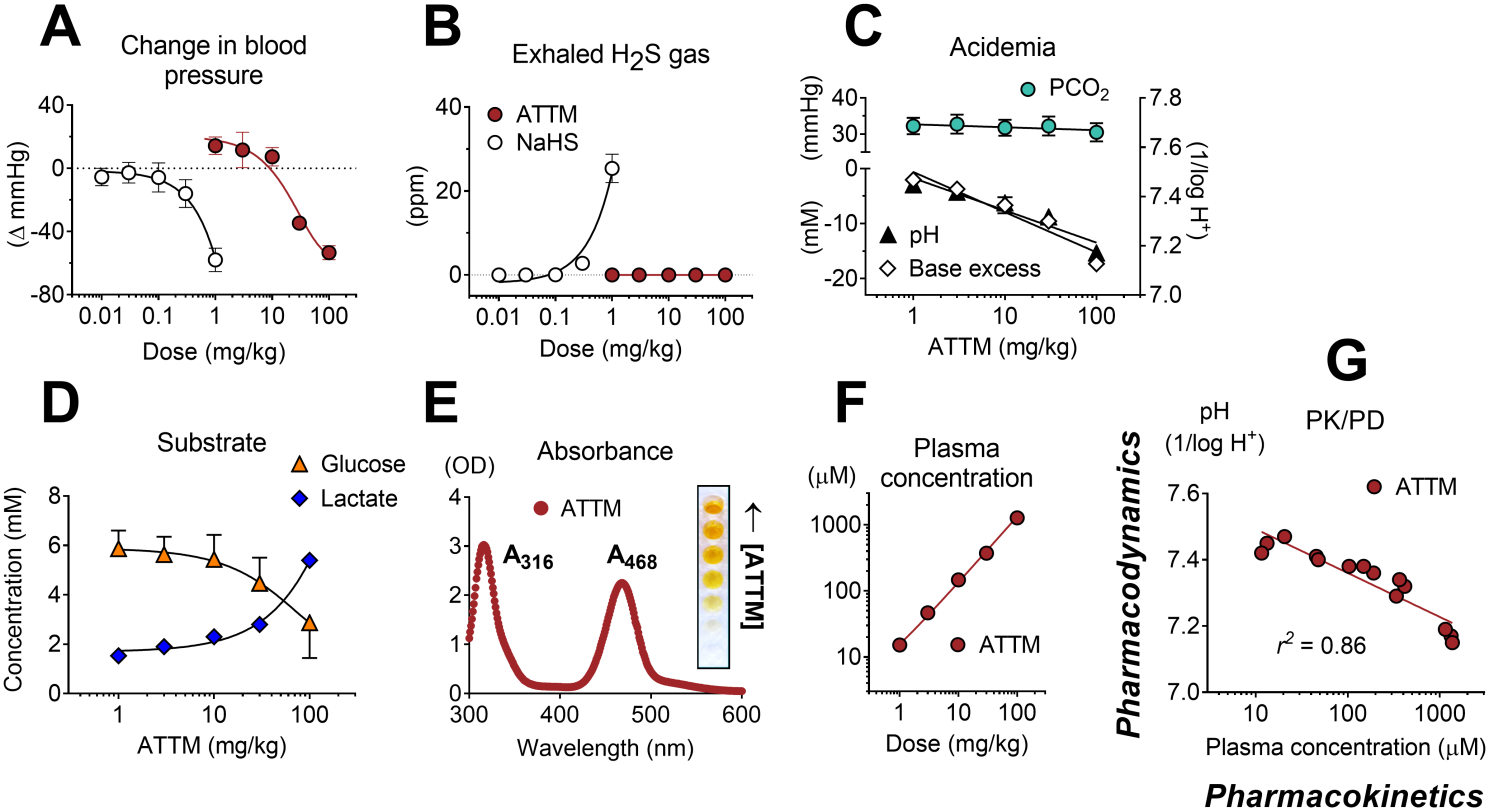
Pharmacokinetic/pharmacodynamic studies. Maximal changes in mean arterial blood pressure (A) and detection of (peak) exhaled H_2_S gas (in parts per million [ppm]) (B) following increasing IV bolus doses of ammonium tetrathiomolybdate (ATTM) or NaHS. No exhaled H_2_S was detectable following ATTM administration. Acid/base interactions following ATTM treatment are shown in (C); the top left *y*-axis denotes (arterial) partial pressure of carbon dioxide (PCO_2_); bottom left and right *y*-axes are (arterial) base excess and pH, respectively. Alterations in (arterial) glucose and lactate following ATTM treatment are shown in (D). (E) shows the absorbance (ultra violet—visible) spectrum of ATTM with (inset) a row of microplate wells used to construct a standard curve. (F) and (G) respectively show changes in ATTM plasma levels (measured using the absorbance peak at 468 nm at 2 min after ATTM administration) against the quantity of drug administered and subsequent (25 min later) changes in arterial pH. *n* = 3–4/group.

To better understand the pharmacological basis of this metabolic modulation, we performed additional pharmacokinetic/pharmacodynamic (PK/PD) studies in anesthetized, mechanically ventilated, and, importantly, normothermic rats. In a dose escalation study, bolus injection of both ATTM and NaHS caused transient (<1 min) hypotension, although far higher (100-fold) doses of ATTM were required to produce an equivalent peak fall in blood pressure, thus demonstrating a superior safety profile (Fig 3A). In animals that received NaHS, significant quantities of H_2_S gas were measured in exhaled breath (Fig 3B). By contrast, and despite the fact that ATTM caused similar hemodynamic effects at the highest dose, no H_2_S gas was detectable in the exhaled breath of animals receiving ATTM, at any dose level. In animals administered ATTM, a dose-dependent metabolic acidemia was observed, while the arterial partial pressure of carbon dioxide (PCO_2_) remained unchanged (Fig 3C). A concurrent increase in blood lactate levels and decrease in glucose (Fig 3D) reflect a greater reliance on non-mitochondrial respiration. Due to the distinct spectral characteristics of ATTM (Fig 3E), plasma levels could be measured using a basic absorbance assay. Plasma concentrations (2 min post-injection) were directly proportional to both the dose administered (Fig 3F) and subsequent (25 min later) changes in arterial pH (Fig 3G), with the latter confirming a strong (*r*^*2*^ = 0.86) and straightforward PK/PD relationship.

Using the same model, we compared the alternative, bis-choline salt of tetrathiomolybdate, known commercially as ATN-224 (Decuprate and WTX101), and found significantly less sulfide release in vitro (S5A Fig; *p <* 0.05). In vivo, the order of effect on arterial pH and lactate (used here as biomarkers of inhibition of oxidative metabolism) was ATTM > ATN-224 > NaHS (S5B and S5C Fig). All three drugs caused comparable degrees of transient hypotension at the highest dose level (S5D Fig), while circulatory (cardiac output) and respiratory (arterial PCO_2_) function remained unchanged (S5E and S5F Fig; *p* = 0.87 and 0.40, respectively). As ATN-224 has the same (molybdenum-based) spectral signature as ATTM, we compared their PK/PD relationships (S5G Fig). ATTM showed a greater fall in arterial pH relative to plasma concentration (slope in S5G Fig), thus reflecting greater potency.

### Sulfhemoglobin formation and safety monitoring

**Fig 4:**
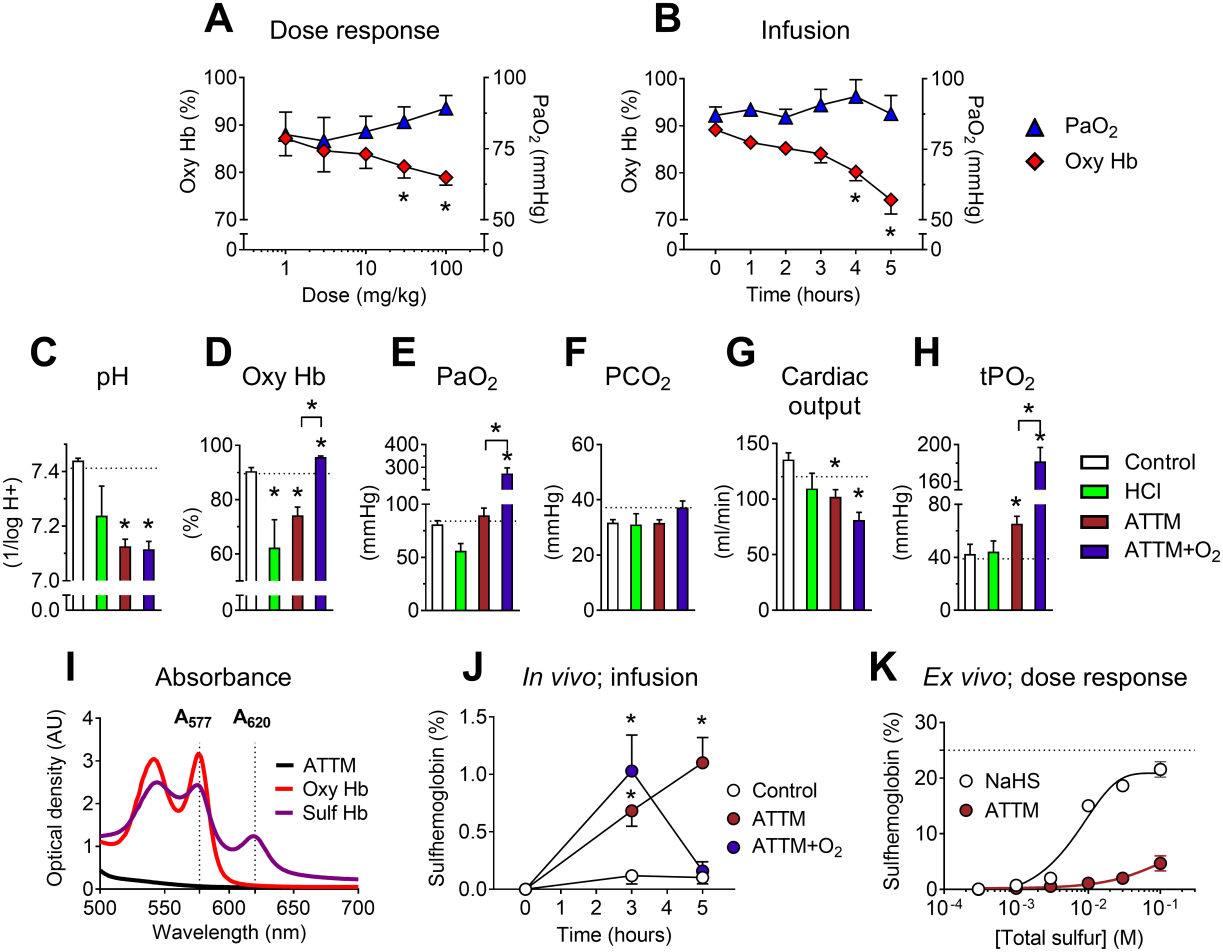
Safety studies. Effects of ammonium tetrathiomolybdate (ATTM) via either IV bolus dosing (A) or continuous infusion (B) on the arterial partial pressure of oxygen (PaO_2_) and percentage of oxygenated hemoglobin (oxy Hb). For the continuous infusion study, changes in acid/base balance, hemodynamics, and muscle tissue oxygen tension (tPO_2_) at experiment end (5 h) are shown in (C–H); dotted lines denote the average baseline value. Where applicable, supplemental oxygen was commenced from 3 h. (I) shows the absorbance spectrum of oxy- and sulfhemoglobin, used for calculation of sulfhemoglobin levels in vivo (in J; infusion study). Note that there was no absorbance overlap between either hemoglobin form and ATTM (1 mM) at λ577/620. Formation of sulfhemoglobin ex vivo using either ATTM or NaHS to spike naïve rat blood is shown in (K). Here, the dotted line represents the maximum sulfhemoglobin level. *p < 0.05 versus baseline (i.e., before the addition of ATTM) in panels A, B, and J using a two-way ANOVA followed by Bonferroni’s testing; *p < 0.05 versus control (and ATTM versus ATTM + O_2_) in panels C–H using a one-way ANOVA followed by Dunn’s multiple comparison test. *n* = 5–10 (in vivo), and *n* = 3 per group (ex vivo) in (K).

In the PK/PD study outlined above, we observed a fall in percent oxyhemoglobin, despite no change in the arterial partial pressure of oxygen (PaO_2_; Fig 4A). We postulated that this observation, a potentially deleterious side effect, could be due to either an acidosis-induced rightward shift of the hemoglobin dissociation curve or the formation of sulfhemoglobin. Given the importance of safety monitoring in drug development, we designed an experiment to address these hypotheses.

An infusion study in anesthetized rats (over 5 h) compared the effects of noncellular acidosis induced by IV hydrochloric acid administration and that induced by high-dose ATTM. We further examined the reversibility of ATTM pharmacology using 70% inspired oxygen (between 3 and 5 h). ATTM infused over 5 h reduced oxyhemoglobin levels (Fig 4B) to a similar magnitude as observed in the PK/PD study (Fig 4A). Continuous infusion of HCl also caused an equivalent fall in arterial pH and oxyhemoglobin (Fig 4C and 4D), consistent with an acidosis-induced rightward shift of the hemoglobin dissociation curve. As expected, an increase in inspired oxygen concentration significantly elevated PaO_2_ (Fig 4E; *p <* 0.01). While this fully reversed the ATTM-induced fall in oxyhemoglobin (Fig 4D), arterial pH remained unchanged (Fig 4C; *p* = 1.0 for animals treated with ATTM versus ATTM + O_2_), and respiratory function remained stable (Fig 4F; *p* = 0.25). Cardiac output decreased with ATTM treatment (Fig 4G; *p <* 0.01), mainly due to a fall in heart rate. Despite this modest yet significant fall in global oxygen delivery, interstitial skeletal muscle tissue oxygen tension (tPO_2_), a marker of the local cellular oxygen supply/demand balance, was elevated (Fig 4H; *p <* 0.05). This is indicative of adequate provision of oxygen, yet decreased utilization by mitochondria.

Sulfhemoglobin levels were measured using an absorbance assay, taking advantage of the differing spectral signatures of oxy- and sulfhemoglobin (Fig 4I). Sulfhemoglobin levels were significantly (*p <* 0.05) elevated after 3 h of ATTM infusion, but fully reversible with increased inspired oxygen (Fig 4J). Far higher drug concentrations were required for pathological sulfhemoglobin formation ex vivo (Fig 4K) than those achieved in the in vivo studies.

### Ischemia/reperfusion models

**Fig 5:**
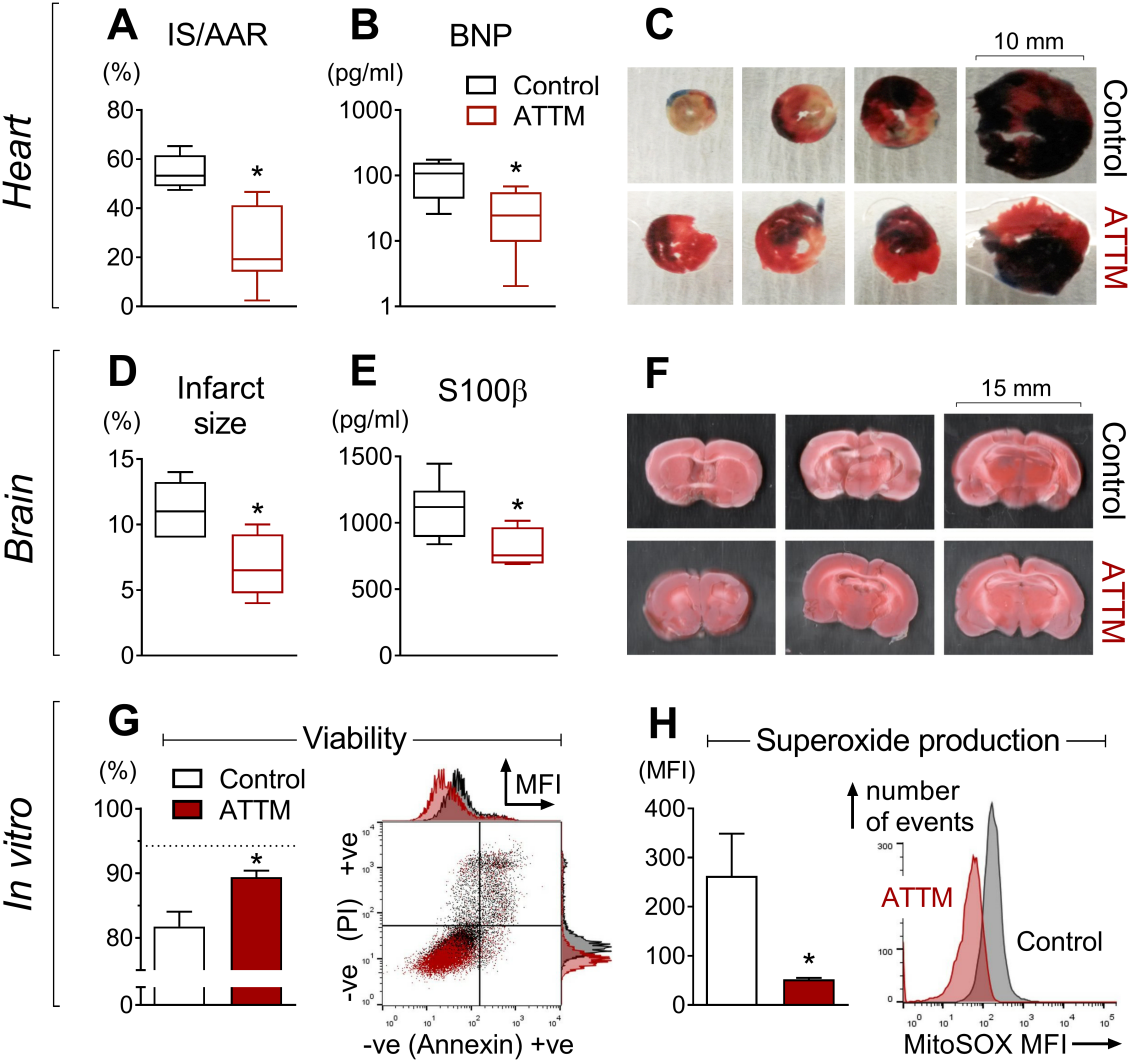
Organ-specific ischemia/reperfusion and cellular anoxia/reoxygenation. Ammonium tetrathiomolybdate (ATTM) confers cardioprotection (A–C, *n* = 6), neuroprotection (D–F, *n* = 6), and cytoprotection (G and H, ATTM 5.5 mM, *n* = 3). In (C), Evans blue dye shows the myocardial area not at risk. In (C) and (F), viable tissue appears pink/red, with non-viable tissue depicted as white/beige. In (G) a representative flow cytometry plot denotes positive (+ve) and negative (−ve) labeling for propidium iodide (PI) and Annexin V, expressed as median fluorescence intensity (MFI). Here, ATTM and controls are depicted in dark red and black, respectively. Live cells (negative for both labels) are shown in the bottom left quadrant and as a percentage of the total in the figure (G, left panel). The dotted line (G, left panel) shows viability in untreated cells (no ischemia/reperfusion or drugs). A representative fluorescence histogram of MitoSOX (mitochondrial superoxide production) is shown in (H), right panel. AAR, area at risk; BNP, B-type natriuretic peptide; IS, infarct size; S100β, S100 calcium binding protein β. *p < 0.05 using an unpaired *t*-test.

The putative efficacy of ATTM was tested both in vitro and in three distinct, clinically relevant in vivo models of I/R injury. In the first two models (organ-specific: heart and brain), ATTM given at reperfusion significantly decreased infarct size (Fig 5A and 5D; *p <* 0.01 and *p* < 0.05, respectively), myocardial-derived B-type natriuretic peptide (Fig 5B; *p <* 0.05), and brain-derived S100 calcium binding protein β (Fig 5E; *p <* 0.05). Representative slices for each model are shown in Fig 5C and 5F. For the heart model, the area at risk as a proportion of the left ventricular area studied was similar across all groups (S6 Fig; *p* = 0.48), demonstrating that the insult applied was of comparable severity.

In our in vitro model, ATTM given at reoxygenation significantly (*p <* 0.05) improved cell viability (Fig 5G) in a concentration-dependent manner (S7A Fig). This was accompanied by a substantial reduction in mitochondrial superoxide production (tested at the highest ATTM concentration, 5.5 mM; Fig 5H). A representative flow cytometry plot (Fig 5G, right panel) and histogram for viability and superoxide production (Fig 5H, right panel) are shown. Incubating cells with ATTM (without I/R) had no effect (*p* = 0.4) on viability (S7B Fig).

**Fig 6:**
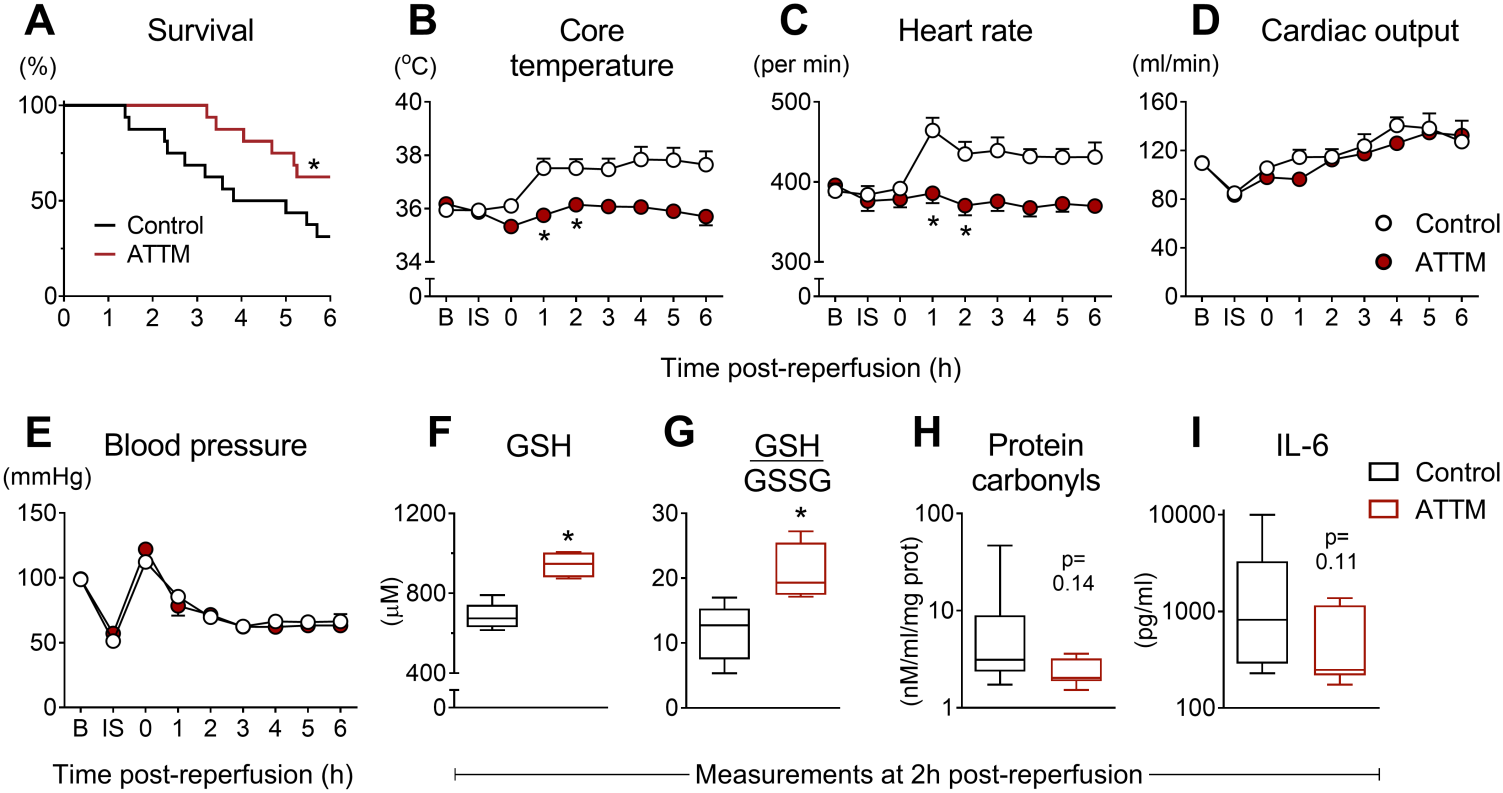
Global ischemia/reperfusion study. Post-reperfusion survival times are shown in (A) for animals treated with ammonium tetrathiomolybdate (ATTM) versus vehicle (control). *p < 0.05, log-rank test, *n* = 16 per group. Sequential changes in core temperature, heart rate, cardiac output, and blood pressure are shown (B–E). *p < 0.05 using a two-way repeated measures ANOVA plus Bonferroni test. Note that this test was only performed up to 2 h post-reperfusion due to early mortality. Measurements of (F) blood levels of reduced glutathione (GSH; antioxidant reserve capacity), (G) the ratio of GSH to oxidized glutathione (GSSG) (lower values indicate greater oxidative stress), (H) protein carbonyls (oxidative damage), and (I) interleukin-6 (IL-6; systemic inflammation) were performed at 2 h post-reperfusion, before the onset of significant mortality. *p < 0.05, unpaired *t*-test.

In our final (global) I/R model, ATTM significantly improved survival time (Fig 6A), such that, at experiment end, twice as many ATTM-treated animals (10/16) were alive compared to the group receiving vehicle (5/16; *p <* 0.05). Following reperfusion, the core temperature and heart rate of vehicle-treated animals increased. ATTM commenced at the time of reperfusion ablated these responses (Fig 6B and 6C; *p <* 0.001 for both) while maintaining global hemodynamics, as evidenced by comparable cardiac output and blood pressure measurements (Fig 6D and 6E; *p* = 0.05 and 0.42, respectively).

Blood taken at 2 h post-reperfusion (before significant mortality) showed significantly (*p <* 0.05) higher levels of GSH, a marker of antioxidant reserve capacity, in ATTM-treated animals (Fig 6F), and a significant (*p <* 0.05) improvement in the ratio of GSH to GSSG, a marker of oxidative stress (Fig 6G). This was accompanied by decreases in oxidative damage (protein carbonyls; Fig 6H) and systemic inflammation (IL-6; Fig 6I); although the point estimates for these biomarkers were different across treatment groups, the differences were not statistically significant (*p* = 0.14 and 0.11, respectively) due to high variability in the control arm.

## Discussion

The last decade has seen considerable interest in sulfide as a putative adjunctive therapy during resuscitation from various acute and critical illness states. Initial studies involved administration of H_2_S gas and simple, inorganic sulfide salts (Na_2_S and NaHS) that, when dissolved, undergo instantaneous “generation” (rather than release) of sulfide [25]. Use of the latter has implications for both safety and efficacy, and most likely precluded the clinical development of treatments with these salts. More sophisticated drug design was clearly needed, and, as such, a diverse range of slow-release sulfide “donors” have been synthesized. These range from phosphinodithioate analogues (e.g., GYY4137), dithiolethiones, and sulfide-hybrid drugs (e.g., S-diclofenac) to persulfide-, *gem*-dithiol-, and *N*-mercapto-based donors [26–33].

Our recognition that ATTM releases sulfide when dissolved in aqueous solution [19] has been historically [34] and more recently [35] hypothesized. In addition, this feature has been informally documented as a side effect (sulfur “burps”) following oral administration of the bis-choline salt, ATN-224, which could be prevented by co-administration of an inhibitor of gastric acid production [36]. Most recently, sulfide release was demonstrated in vitro; Xu et al. reported that ATTM acts as a water-soluble, slow-release sulfide donor that is pH-sensitive [37].

ATTM is not formally known as either a sulfide generator or a sulfide donor in the medical literature. Although it shares characteristics of both, using recently updated nomenclature [25], it is neither. Similarities between ATTM and the sulfide generators relate to their inorganic structure and sensitivity to pH in aqueous solution. However, whereas the simple salts release all sulfide when dissolved, ATTM releases sulfide in a linear fashion over time at physiological pH and temperature, with increased sulfide release favored in warmer, more acidic conditions. Sulfide release from all putative donors relies on some form of activation, with mechanisms including hydrolysis, light, pH, and thiol activation. Surprisingly, combinations of these factors that affect sulfide release are rarely addressed [26,37]. That ATTM releases sulfide spontaneously in aqueous solution in a pH-sensitive manner is indicative of hydrolysis; however, we further found that ATTM can be thiol-activated in vitro. To our knowledge, no sulfide donors activated by hydrolysis have shown additional sensitivity to biologically relevant thiols. This has clear consequences for sulfide release in vivo, where thiols (particularly albumin in plasma and GSH intracellularly) may be present at levels capable of catalyzing this reaction. In summary, our data demonstrate (1) that there are distinct differences in the nature of sulfide release from NaHS and ATTM and (2) that the release of sulfide from ATTM differs from that of other previously described sulfide-releasing drugs, being activated by a range of chemical and biologically relevant factors. Taken together with the chemical characteristics of ATTM, our findings position this molecule within a new class of sulfide-releasing drugs.

Our particular interest in sulfide relates to its ability to modulate metabolism and the implications this could have for the treatment of I/R injury. While the therapeutic potential of hydrogen sulfide is well established, it is also toxic at higher concentrations [38], and this restricts its use in patients. It modulates metabolism by potent inhibition of mitochondrial cytochrome C oxidase (complex IV), thereby inhibiting oxidative phosphorylation and aerobic generation of adenosine triphosphate (ATP) [39]. We compared the biological activity of NaHS and ATTM ex vivo; while both could inhibit oxygen consumption, unsurprisingly NaHS exhibited greater potency by its rapid generation of sulfide. A recent study using a hypoxic cell model demonstrated that ATTM could inhibit complex IV, though this was assumed to be mediated via a copper-dependent pathway involving hypoxia-inducible factor 1-alpha [40]. We, too, found that oxygen consumption ex vivo was dependent on the local oxygen concentration. However, given the timeframe of action (seconds to minutes) in our experiments, plus the lack of inhibitory activity of other non-sulfide-containing copper chelators, we contend that the dependence of drug activity on local oxygen concentration relates to reduced oxidation of sulfide under hypoxic conditions rather than an inducible or copper-dependent biological mechanism.

In vivo, we found that the side effects of NaHS were dose-limiting, with a maximum tolerated (IV bolus) dose of 1 mg/kg. By contrast, far higher doses of ATTM (up to 100 mg/kg) could be tolerated, despite exposure to a greater concentration of a sulfide-containing drug with a substantially longer half-life. Using intermediate doses in awake rats allowed us to safely reduce the whole body metabolic rate by 25%. Strikingly, in this model we observed that heart rate fell by one-third, a pharmacological response that would considerably decrease myocardial oxygen demand, yet without obvious injury or detriment to the animal.

To better understand the impact of ATTM on global metabolism in vivo, we undertook detailed PK/PD studies. A normothermic model ensured that responses were of a primary pharmacological nature, and not confounded by secondary responses such as hypothermia. Increasing IV bolus doses of ATTM caused a dose-dependent metabolic acidemia that directly correlated with plasma concentrations. This, as well as the reduction in oxygen consumption, supports our proposed intracellular mechanism of action at the level of the mitochondrion; when the lungs and/or circulation cannot deliver, or the mitochondria cannot utilize, sufficient quantities of oxygen to sustain oxidative metabolism, cells increase both ATP hydrolysis and glycolysis, both of which generate an excess of protons, and thus acidemia. The concurrent fall in blood glucose and rise in lactate also support this shift in metabolism towards glycolysis following inhibition of oxidative phosphorylation. The lack of significant change in arterial partial pressure of carbon dioxide and global oxygen delivery, and the rise in skeletal muscle tissue oxygen tension, indicates ongoing cardiorespiratory function that can still supply sufficient oxygen and substrate to adequately meet (albeit reduced) metabolic demand [41].

We formally demonstrated that the bis-choline salt of tetrathiomolybdate, ATN-224, releases sulfide in vitro, though to a lesser extent than ATTM. This feature explains its comparative impotency in vivo with regards to acidemia and other secondary markers of mitochondrial inhibition. For ATN-224, this is a desirable feature; it is being developed as an oral agent for use in Wilson disease and cancer [18,42]. Its copper-binding properties in the gastrointestinal tract (copper is required for angiogenesis) require the molecule to remain intact for chelation [43]. These data demonstrate that sulfide-releasing thiometallate drugs extend beyond ATTM, that they vary in terms of their magnitude of sulfide release, and that their in vivo potency can be predicted by a simple in vitro test.

ATTM has a good safety record in humans, having been used orally over long periods as a copper chelating therapy (albeit off-label) for Wilson disease [15]. There are known side effects related to copper depletion, most notably hematological events such as pancytopenia and elevations in liver enzymes. However, these side effects have been documented only after chronic administration, and they respond well to either dose reduction or drug holiday [18]. ATTM has been assessed intravenously in a small series of patients with Wilson disease with no reported safety concerns [17]. Although not addressed in the current study, we do not anticipate copper-related safety concerns with a single IV dose of ATTM given at reperfusion, as it takes several weeks of therapy to deplete systemic copper levels [18].

As noted above, the nature of sulfide release from any putative medicine has implications for safety and efficacy. Given that hydrogen sulfide has historically been regarded as an environmental poison, with numerous reports of near-fatal intoxication [38,44,45], we considered safety monitoring to be of particular importance in our study. As our preclinical studies indicated utility for ATTM as an IV therapy against reperfusion injury, we were keen to address hypotension and sulfhemoglobin formation in further detail, as these may represent limiting complications. Sulfide-induced vasodilation is well recognized, with putative mechanisms including covalent modification (sulfhydration) of the K_ATP_ channel, an inability to maintain vascular tone due to ATP depletion, intracellular acidification, modulation of calcium signaling, and release of lipid mediators [7]. NaHS, ATTM, and ATN-224 all caused transient hypotension when given by bolus IV injection, though (compared to NaHS) far higher doses (100-fold) of the thiometallates were required to elicit an equivalent blood pressure effect in our healthy animals. While NaHS produced detectable levels of exhaled H_2_S gas (indicative of free circulating sulfide), this outcome was not observed with ATTM or ATN-224 at any dose level. This suggests that thiometallates release the majority of their sulfide within an extravascular compartment. This is likely to occur intracellularly, where the microenvironment is more acidic and higher levels of thiols are present. The intense absorbance characteristics of the molecule, while convenient for plasma determination, precluded affirmation of this postulate using flow cytometry with fluorescent probes. Notwithstanding this lack of confirmation, the putative extracirculatory sulfide release generated far greater cellular metabolic potency in vivo, i.e., increased acidemia for the same level of hypotension, thus demonstrating that, in our animal model, targeted cellular delivery permits a superior safety window for ATTM over rapid sulfide donors or generators.

The consistent fall in oxyhemoglobin saturation with ATTM, despite maintenance of arterial oxygen partial pressure, suggested possible formation of sulfhemoglobin. While we could generate significant sulfhemoglobin ex vivo, far higher concentrations of free sulfide (low millimolar range) were required than we could encounter in vivo (micromolar range). Moreover, high-dose administration of ATTM did generate a 1% sulfhemoglobin level in vivo, but this does not account for the 15% fall in oxyhemoglobin saturation. A pH-induced right shift of the hemoglobin dissociation curve is more likely, as reversal was seen following administration of high oxygen concentrations.

Since elimination of sulfide is predominantly believed to be due to oxidation (largely occurring in the mitochondria), we speculated that administration of oxygen could ameliorate all of the pharmacological effects of ATTM and, in essence, offer an easily applied antidote. However, while the fall in oxyhemoglobin saturation and the low level sulfhemoglobin could be reversed, arterial pH remained unchanged. This is in keeping with the proposed nature of sulfide binding at complex IV; although widely considered to be a reversible process, with sulfide competing with oxygen in a similar manner to nitric oxide and carbon monoxide, detailed biochemical experiments (albeit using purified proteins) revealed that inhibition is noncompetitive with respect to cytochrome C and oxygen [6,46]. While this could explain the inability to reverse the acidosis by high inspired oxygen concentrations, mice exposed to H_2_S gas made a rapid, complete recovery upon cessation of administration [5]. It is unclear at present whether the temporal nature of this recovery is species-specific.

Efficacy studies are the mainstay for preclinical proof of concept. Sulfide generators and donors have shown a good track record in preclinical I/R studies; numerous reports have demonstrated cardioprotection, neuroprotection, and outcome benefit [9,47,48]. Mechanisms postulated include preservation of mitochondrial function, modulation of inflammation and apoptosis, enhanced angiogenesis, and reduced oxidative stress [7,13]. While secondary tissue-specific pathways could confer benefit within particular organs, these pathways most likely originate from an initial trigger. Both ischemia and reperfusion phases generate ROS that can subsequently damage lipids, proteins, and DNA, and the majority of ROS derive from mitochondria [1]. During ischemia, the vitality of cells in the area at risk will be either irretrievable or salvageable (closer to the surrounding penumbra). On revascularization, salvageable cells are subjected to far greater restoration of nutrient supply than their metabolic state requires, with enhanced substrate provision driving oxidative phosphorylation and generating large quantities of ROS. We have shown efficacy in three distinct, clinically relevant in vivo I/R models that supports a universal mechanism of action: that delivering a therapy to modulate mitochondrial function decreases ROS production. This would be expected, as observed, to enhance antioxidant reserve capacity and reduce oxidative stress and damage. The mechanistic measures in our global I/R model and our in vitro I/R study support these claims. Additional benefit may derive from a direct sulfide ROS scavenging effect and/or indirect effects such as increased intracellular acidity enabling more rapid reduction of GSSG back to GSH [49]. Lastly, ischemic cells are more likely to exhibit decreased pH levels. Since sulfide release from ATTM is pH-dependent, more sulfide may be released within cells that are more acidic and therefore have a greater need for treatment.

We demonstrated a beneficial effect for ATTM as a novel adjunct therapy for myocardial and cerebral I/R injury, but its use in other emergency settings where modulation of metabolism can potentially confer benefit also merits exploration. We found survival benefit when ATTM was given at reperfusion following severe hemorrhage, and a previous study also demonstrated that rats could tolerate greater blood loss when given a basic sulfide salt [8]. Preclinical efficacy is also reported with sulfide in traumatic brain injury models [50,51].

### Limitations

It is noteworthy for future study that not all sulfide detection assays are suitable for pH-sensitive sulfide-releasing molecules such as ATTM. Xu et al. used a modified methylene blue assay, mindful of the fact that pH alters sulfide release from this molecule [37]. In addition, our initial pharmacokinetic comparison studies between ATTM and NaHS showed that certain fluorophores and/or additives can potentially interact with the molecule. As such, we documented high plasma levels of sulfide (mid-micromolar range) following ATTM treatment that, as free sulfide, would not be compatible with life. We consider the majority of sulfide to be still bound to the molecule and not bioavailable within the circulation. Since we used cysteine as an adjunct to bind excess derivatizing agent, covalently bound sulfide would be made available by thiol-induced release during sample processing with our modified MBB assay. This, and the fact that the MBB reaction may detect additional reactive sulfur species [21], means that the MBB and headspace H_2_S gas methods employed here reflect total and free sulfide, respectively. Also noteworthy of consideration is the ATTM manufacturer. We found good consistency across batches from one source but would urge other researchers to establish and standardize sulfide release assays, if used for this purpose. Unfortunately, coloration (and consequent absorption of incident light) of the molecule precluded measurement of intracellular sulfide levels by flow cytometry using fluorescence probes such as WSP-1 [52] and POMAL-N3 [53]. Its coloration could also interfere with other absorption techniques such as the oximetry component of arterial blood gas analysis and, potentially, the measurement of oxyhemoglobin saturation. Penultimately, although animals in our awake, tethered model appeared clinically normal with a 24-h infusion of ATTM, we did not perform any comprehensive behavioral testing nor long-term follow-up. This will be addressed in future (certified) toxicology, safety, and tolerability studies. Lastly, we acknowledge that our work is exclusively of a preclinical nature. To this end, rigorous human testing will be additionally required to establish the safety of ATTM (by IV administration) and its therapeutic utility for reperfusion injury in a clinical setting.

### Conclusion and development plan

Although the sulfide generators have so far fallen short of becoming new medicines, there is precedent for reduced metabolism conferring protection in I/R injury in humans. For example, an outcome benefit has been demonstrated from therapeutic hypothermia in various situations, including out-of-hospital cardiac arrest [54] and during myocardial revascularization [55]. However, as this procedure is cumbersome and target temperatures are often not achieved for 4–6 h, it has not proven universally beneficial [56]. ATTM could offer a safe, rapid, and easily applied alternative. Importantly, it has a good safety record in humans that includes IV use in patients [17], thus providing much encouragement for translation of this compound to the clinical arena as a novel adjunct treatment for reperfusion injury. Our primary target is acute myocardial infarction, where ATTM could be delivered intravenously or directly into the coronary circulation at the time of angioplasty.

## Supporting information

S1 Arrive Guidelines A checklist providing reference to all aspects of animal experimentation performed in this study. (DOCX)

S1 Fig In vitro H_2_S gas released from different batches of ATTM. Batches 1–5 were from the same commercial supplier, though from different manufacturer “lots.” No H_2_S gas was detectable from batch 6, purchased elsewhere. The dotted lines reflect typical H_2_S gas levels obtained from ATTM (100 mM total sulfur) following 1 h incubation at normal physiological pH and temperature (3–4 ppm). *n* = 3/group.
(TIF)

S2 Fig In vitro sulfide release using a monobromobimane-based assay. Sulfide release from ATTM and NaHS at varying pH is shown in (A) and (B), respectively. Samples were incubated at 37°C. ATTM, 0.55 mM, and NaHS, 2.2 mM, were used to give equimolar sulfur concentrations, as indicated by the dashed lines. Note that in our modified version of this assay we added L-cysteine to bind excess MBB. This induces thiol-dependent release of sulfide from ATTM. As such, between pH 4.5 and 10, ATTM releases approximately 80% of its bound sulfur as sulfide under these conditions. *n* = 4/group.
(TIF)

S3 Fig Plasma sulfide concentrations following IV dosing in vivo. Monobromobimane-derived plasma sulfide concentrations in rats receiving a bolus IV injection (A) or continuous infusions (B) of ATTM. Bolus IV injections of ATTM and NaHS with comparable total sulfur are shown in (C) to illustrate their fast “distribution” decay curves. (D) shows comparative pharmacokinetics for ATTM and NaHS following IV bolus dosing. The slow “elimination” half-life of NaHS was not determined (n/d) as plasma concentrations rapidly normalized. *n* = 3–4 per group.
(TIF)

S4 Fig Effects of non-sulfide-containing copper chelators on oxygen consumption in rat soleus muscle ex vivo. Experiments were performed in (A) normoxia (150–250 μM O_2_) or (B) hypoxia. In (A), increasing concentrations of combined neocuproine and cuprizone treatment were used. In (B), combined treatment at a single concentration (100 μM of each) was added at 200 μM O_2_, and tissues allowed to respire to hypoxia. Two-way repeated measures ANOVA plus Bonferroni’s test revealed no significant differences between control (respiratory medium) and copper chelator treatment. *n* = 8 per group.
(TIF)

S5 Fig Comparative pharmacological assessment of sulfide-releasing drugs. (A) shows significant differences in H_2_S gas release in vitro from ATTM and ATN-224. *p < 0.05, unpaired *t*-test, *n* = 3. Dashed lines represent standard release under these conditions (3–4 parts per million [ppm] after 60 min incubation at pH 7.4 and 37°C). In vivo changes in acid/base balance and hemodynamics at experiment end are shown in (B–F), i.e., following the highest dose of each drug. Here, dashed lines represent the average baseline values obtained. *p < 0.05 compared to ATTM, one-way ANOVA plus Dunn’s multiple comparison test. (G) shows the PK/PD relationships for ATTM and ATN-224. The slope represents the potency of each drug. Since an absorbance assay was used here to determine plasma drug levels, the comparative effects of (non-colored) NaHS were not determined. *n* = 3–4 per group.
(TIF)

S6 Fig Area at risk as a proportion of total area (left ventricle) following myocardial ischemia/reperfusion injury. *p* = 0.48 using an unpaired *t*-test, *n* = 6/group.
(TIF)

S7 Fig In vitro cell studies. Concentration-dependent improvement in viability following I/R with ATTM treatment (A) and no change in viability in normoxic cells (B). In (B), cells were plated and maintained in a normoxic environment for 24 h, then treated with ATTM (5.5 mM) prior to analysis. *p < 0.05 compared to vehicle (shown in Fig 5), one-way ANOVA plus Dunn’s multiple comparison test.
(TIF)

S1 Text A more detailed description of the following methods: monobromobimane assay, in vivo surgical instrumentation, ex vivo metabolic study, and echocardiography. (DOCX)

## Abbreviations

ATP: adenosine triphosphate
ATTM: ammonium tetrathiomolybdate
GSH: reduced glutathione
GSSG: oxidized glutathione
HPLC: high-performance liquid chromatography
I/R: ischemia/reperfusion
IV: intravenous
LAD: left anterior descending
MBB: monobromobimane
PK/PD: pharmacokinetic/pharmacodynamic
ppm: parts per million
ROS: reactive oxygen species
