# Supplementary material for: Ammonium tetrathiomolybdate following ischemia/reperfusion injury: Chemistry, pharmacology, and impact of a new class of sulfide donor in preclinical injury models
Source: PLoS Med. 2017 Jul 5;14(7):e1002310. doi: 10.1371/journal.pmed.1002310 (PMC5497958; doi:10.1371/journal.pmed.1002310)
Supplement: S1 Arrive Guidelines — (DOCX) [file pmed.1002310.s001.docx]

**The ARRIVE Checklist**

TITLE

1 Provide as accurate and concise a description of the content of the article as possible.

Lines 1-3.

ABSTRACT

2 Provide an accurate summary of the background, research objectives (including details of the species or strain of animal used), key methods, principal findings, and conclusions of the study.

Lines 31-46. The species is included the species in the abstract.

INTRODUCTION

Background

3 a. Include sufficient scientific background (including relevant references to previous work) to understand the motivation and context for the study, and explain the experimental approach and rationale.

b. Explain how and why the animal species and model being used can address the scientific objectives and, where appropriate, the study’s relevance to human biology.

1. Lines 93-135 – manuscript introduction.
2. Lines 139-143.

Objectives

4 Clearly describe the primary and any secondary objectives of the study, or specific hypotheses being tested.

Lines 132-135.

METHODS

Ethical statement

5 Indicate the nature of the ethical review permissions, relevant licenses (e.g. Animal [Scientific Procedures] Act 1986), and national or institutional guidelines for the care and use of animals, that cover the research.

Lines 143-147.

Study design

6 For each experiment, give brief details of the study design, including:

1. The number of experimental and control groups.

Lines 179-301.

1. Any steps taken to minimise the effects of subjective bias when allocating animals to treatment (e.g., randomisation procedure) and when assessing results (e.g., if done, describe who was blinded and when).

Lines 318-324.

c. The experimental unit (e.g. a single animal, group, or cage of animals).

A time-line diagram or flow chart can be useful to illustrate how complex study designs were carried out.

Lines 179-301. Group sizes indicated throughout this section where a single animal represents an experimental unit.

Experimental procedures

7 For each experiment and each experimental group, including controls, provide precise details of all procedures carried out. For example:

1. How (e.g., drug formulation and dose, site and route of administration, anaesthesia and analgesia used [including monitoring], surgical procedure, method of euthanasia). Provide details of any specialist equipment used, including supplier(s).

Lines 179-301.

Further detail provided in Supporting Information (S1 Text; surgical instrumentation).

1. When (e.g., time of day).

Line 152.

1. Where (e.g., home cage, laboratory, water maze).

Line 149-151.

d. Why (e.g., rationale for choice of specific anaesthetic, route of administration, drug dose used).

Detail provided in Supporting Information (S1 Text; surgical instrumentation).

Experimental animals

8 a. Provide details of the animals used, including species, strain, sex, developmental stage (e.g., mean or median age plus age range), and weight (e.g., mean or median weight plus weight range).

Line 140.

b. Provide further relevant information such as the source of animals, international strain nomenclature, genetic modification status (e.g. knock-out or transgenic), genotype, health/immune status, drug- or test naıve, previous procedures, etc.

Lines 147-149.

Housing and husbandry

9 Provide details of:

1. Housing (e.g., type of facility, e.g., specific pathogen free (SPF); type of cage or housing; bedding material; number of cage companions; tank shape and material etc. for fish).

Lines 149-152.

1. Husbandry conditions (e.g., breeding programme, light/dark cycle, temperature, quality of water etc. for fish, type of food, access to food and water, environmental enrichment).

Lines 149-152.

c. Welfare-related assessments and interventions that were carried out before, during, or after the experiment.

Lines 155-157, 209-210, 287-289.

Sample size

10 a. Specify the total number of animals used in each experiment and the number of animals in each experimental group.

b. Explain how the number of animals was decided. Provide details of any sample size calculation used.

c. Indicate the number of independent replications of each experiment, if relevant.

Lines 179-301 – see Methodology for sample sizes.

Sample sizes are based on >20 years’ experience.

Allocating animals to experimental groups

11 a. Give full details of how animals were allocated to experimental groups, including randomisation or matching if done.

b. Describe the order in which the animals in the different experimental groups were treated and assessed.

1. Refer to Q6.
2. Lines 319-324.

Experimental outcomes

12 Clearly define the primary and secondary experimental outcomes assessed (e.g., cell death, molecular markers, behavioural changes).

Lines 179-301 in Methodology.

Lines 377-550 in Results.

Statistical methods

13 a. Provide details of the statistical methods used for each analysis.

b. Specify the unit of analysis for each dataset (e.g. single animal, group of animals, single neuron).

c. Describe any methods used to assess whether the data met the assumptions of the statistical approach.

Lines 318-336.

RESULTS

Baseline data
14 For each experimental group, report relevant characteristics and health status of animals (e.g., weight, microbiological status, and drug- or test-naıve) before treatment or testing (this information can often be tabulated).

Lines 140, 149-150.

Numbers analysed

15 a. Report the number of animals in each group included in each analysis. Report absolute numbers (e.g. 10/20, not 50%).

b. If any animals or data were not included in the analysis, explain why.

1. Refer to Q10.
2. Lines 276-279.

Outcomes and estimation

16 Report the results for each analysis carried out, with a measure of precision (e.g., standard error or confidence interval).

Refer to Q12-13.

Adverse events

17 a. Give details of all important adverse events in each experimental group.

b. Describe any modifications to the experimental protocols made to reduce adverse events.

1. Lines 527-529. Our global ischemia/reperfusion model was designed to induce significant mortality under terminal anesthesia.
2. Lines 287-289.

DISCUSSION

Interpretation/scientific implications

18 a. Interpret the results, taking into account the study objectives and hypotheses, current theory, and other relevant studies in the literature.

b. Comment on the study limitations including any potential sources of bias, any limitations of the animal model, and the imprecision associated with the results.

c. Describe any implications of your experimental methods or findings for the replacement, refinement, or reduction (the 3Rs) of the use of animals in research.

1. Refer to Q2 and Q4.
2. Lines 702-726.
3. Lines 209-210 – most our animal studies are non-recovery. Animals that do recover are frequently assessed using an in-house scoring system developed and validated by the authors’ and biological services staff.

Generalisability/translation

19 Comment on whether, and how, the findings of this study are likely to translate to other species or systems, including any relevance to human biology.

Lines 140-143, 723-726.

Funding

20 List all funding sources (including grant number) and the role of the funder(s) in the study.

Funding support comes from Magnus Oxygen and the Medical Research Council Newton Fund (MR/N007085/1) for MS and FDP. FDP further acknowledges FAPESC (Fundação de Amparo à Pesquisa e Inovação do Estado de Santa Catarina). MF acknowledges support from the Medical Research Council (G1001536). MS is a Senior Investigator of the UK National Institute of Health Research (NIHR). UCLH/UCL receives a proportion of funding from the UK Department of Health’s NIHR Biomedical Research funding scheme.

The funders had no role in study design, data collection and analysis, decision to publish, or preparation of the manuscript.
