# Supplementary figures and images for: Ammonium tetrathiomolybdate following ischemia/reperfusion injury: Chemistry, pharmacology, and impact of a new class of sulfide donor in preclinical injury models
Source: PLoS Med. 2017 Jul 5;14(7):e1002310. doi: 10.1371/journal.pmed.1002310 (PMC5497958; doi:10.1371/journal.pmed.1002310)

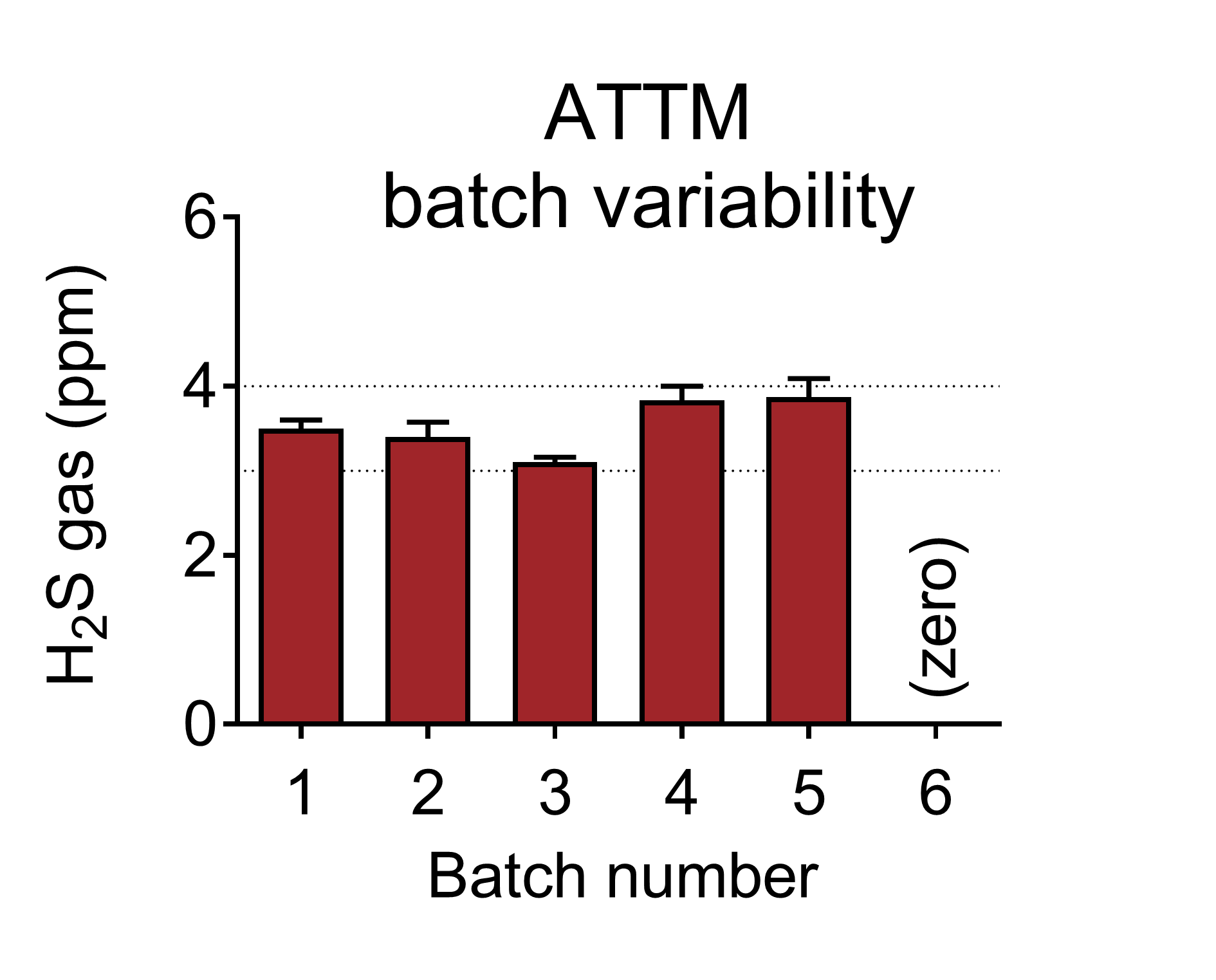

Supplement: S1 Fig — Batches 1–5 were from the same commercial supplier, though from different manufacturer “lots.” No H2S gas was detectable from batch 6, purchased elsewhere. The dotted lines reflect typical H2S gas levels obtained from ATTM (100 mM total sulfur) following 1 h incubation at normal physiological pH and temperature (3–4 ppm). n = 3/group. (TIF) [file pmed.1002310.s002.tif]

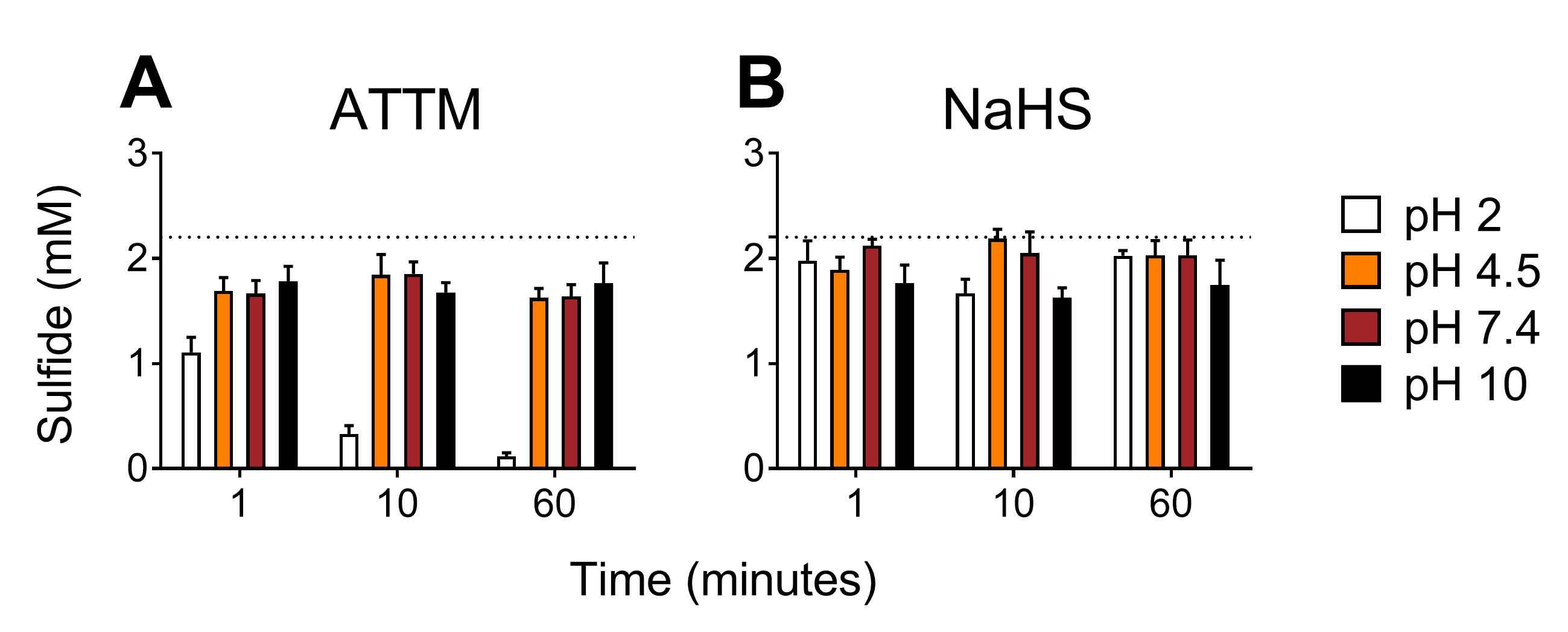

Supplement: S2 Fig — Sulfide release from ATTM and NaHS at varying pH is shown in (A) and (B), respectively. Samples were incubated at 37°C. ATTM, 0.55 mM, and NaHS, 2.2 mM, were used to give equimolar sulfur concentrations, as indicated by the dashed lines. Note that in our modified version of this assay we added L-cysteine to bind excess MBB. This induces thiol-dependent release of sulfide from ATTM. As such, between pH 4.5 and 10, ATTM releases approximately 80% of its bound sulfur as sulfide under these conditions. n = 4/group. (TIF) [file pmed.1002310.s003.tif]

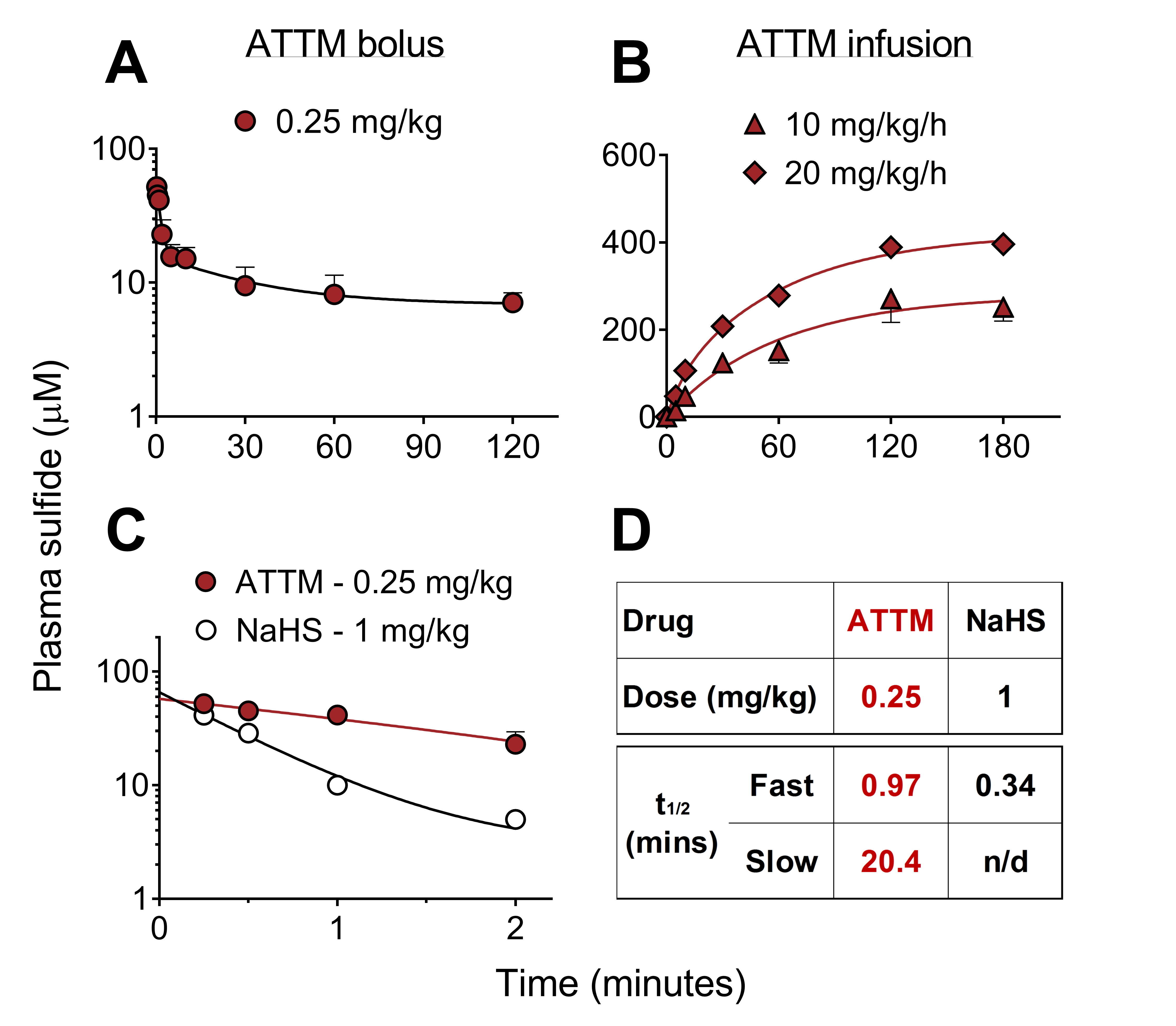

Supplement: S3 Fig — Monobromobimane-derived plasma sulfide concentrations in rats receiving a bolus IV injection (A) or continuous infusions (B) of ATTM. Bolus IV injections of ATTM and NaHS with comparable total sulfur are shown in (C) to illustrate their fast “distribution” decay curves. (D) shows comparative pharmacokinetics for ATTM and NaHS following IV bolus dosing. The slow “elimination” half-life of NaHS was not determined (n/d) as plasma concentrations rapidly normalized. n = 3–4 per group. (TIF) [file pmed.1002310.s004.tif]

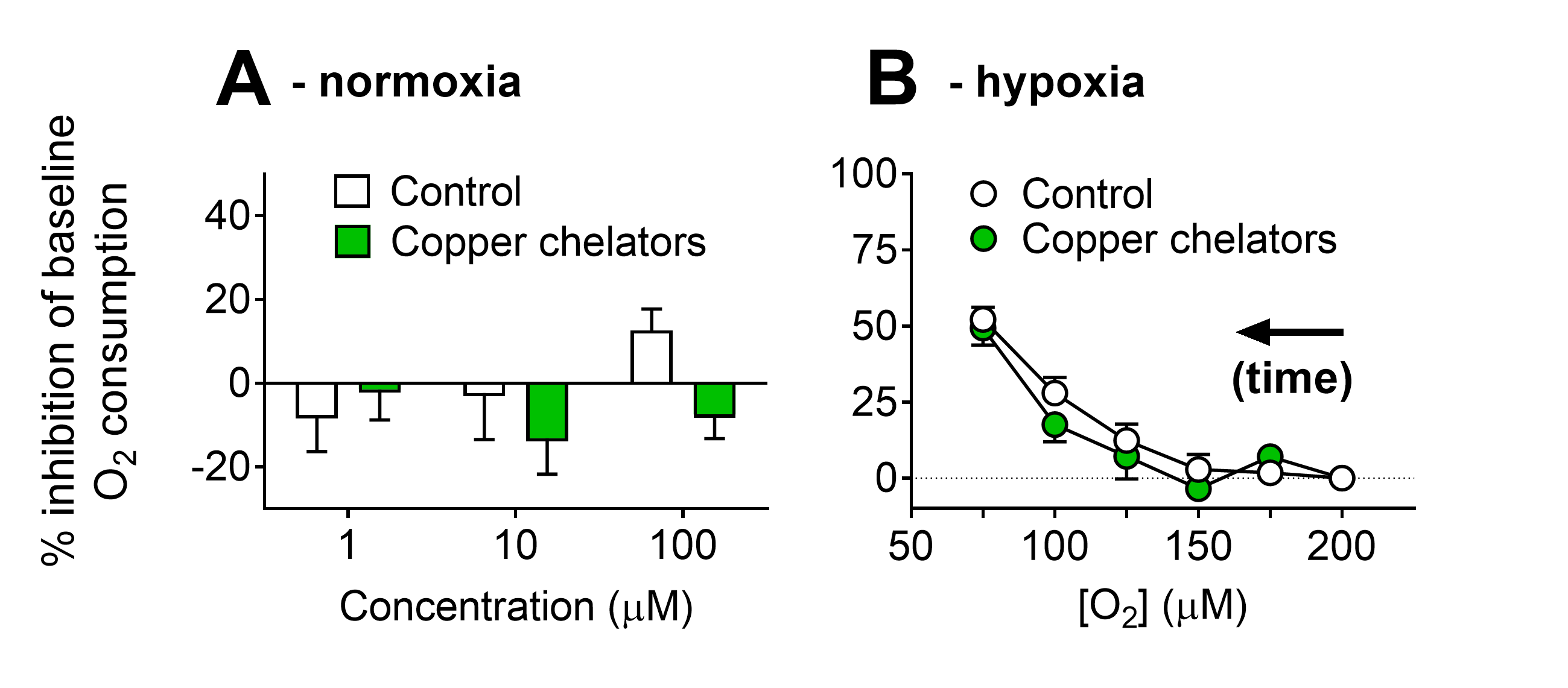

Supplement: S4 Fig — Experiments were performed in (A) normoxia (150–250 μM O2) or (B) hypoxia. In (A), increasing concentrations of combined neocuproine and cuprizone treatment were used. In (B), combined treatment at a single concentration (100 μM of each) was added at 200 μM O2, and tissues allowed to respire to hypoxia. Two-way repeated measures ANOVA plus Bonferroni’s test revealed no significant differences between control (respiratory medium) and copper chelator treatment. n = 8 per group. (TIF) [file pmed.1002310.s005.tif]

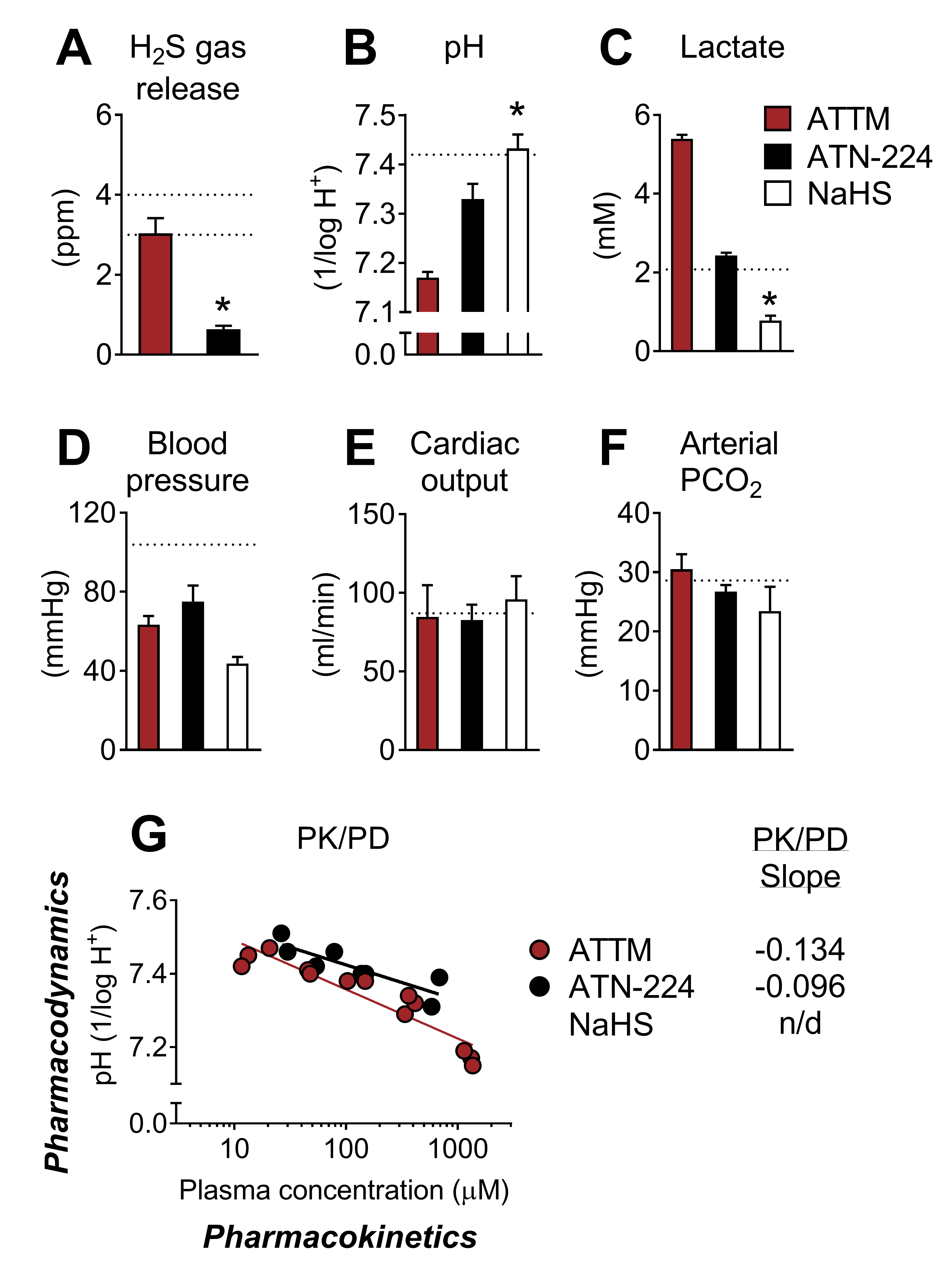

Supplement: S5 Fig — (A) shows significant differences in H2S gas release in vitro from ATTM and ATN-224. *p < 0.05, unpaired t-test, n = 3. Dashed lines represent standard release under these conditions (3–4 parts per million [ppm] after 60 min incubation at pH 7.4 and 37°C). In vivo changes in acid/base balance and hemodynamics at experiment end are shown in (B–F), i.e., following the highest dose of each drug. Here, dashed lines represent the average baseline values obtained. *p < 0.05 compared to ATTM, one-way ANOVA plus Dunn’s multiple comparison test. (G) shows the PK/PD relationships for ATTM and ATN-224. The slope represents the potency of each drug. Since an absorbance assay was used here to determine plasma drug levels, the comparative effects of (non-colored) NaHS were not determined. n = 3–4 per group. (TIF) [file pmed.1002310.s006.tif]

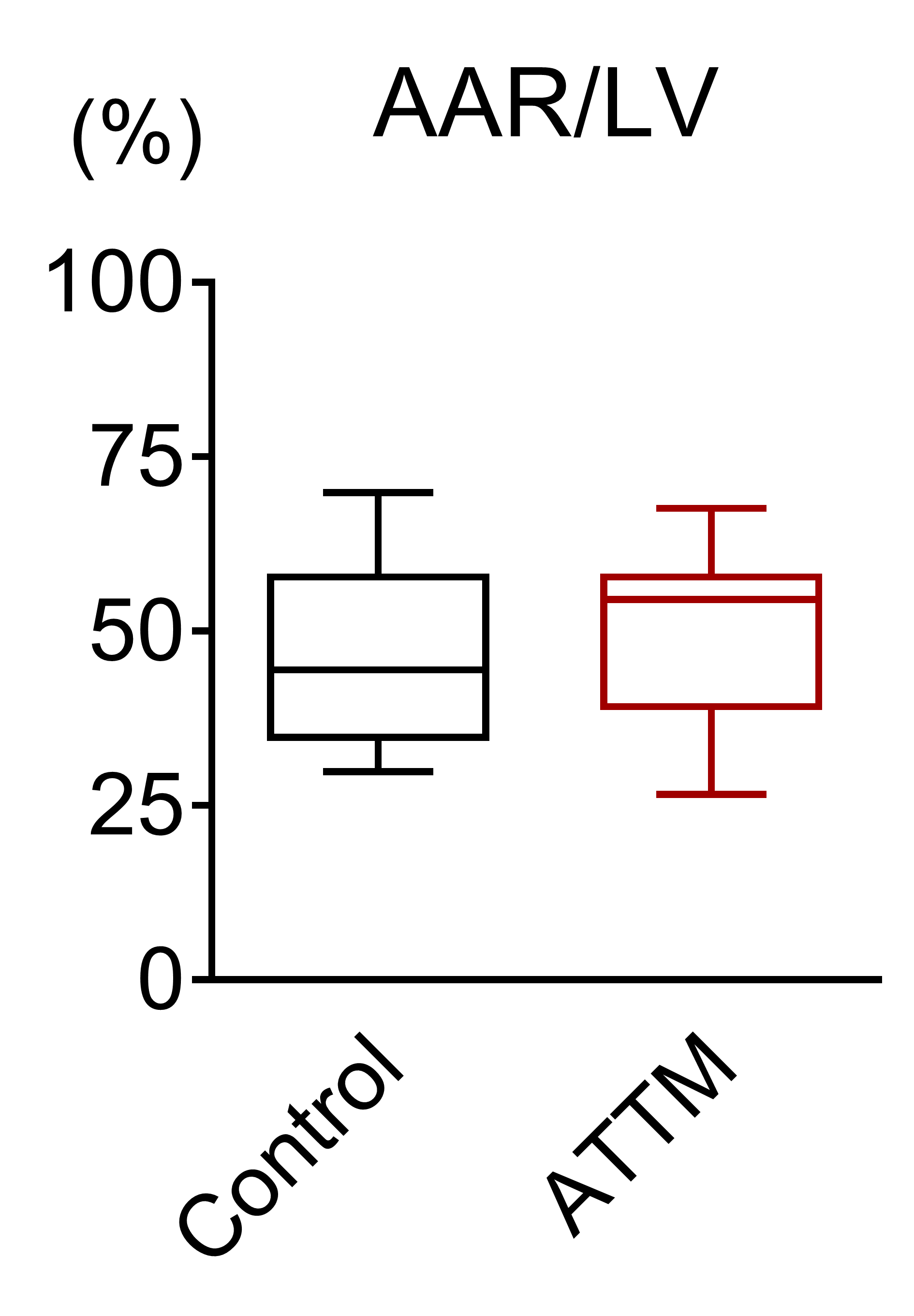

Supplement: S6 Fig — p = 0.48 using an unpaired t-test, n = 6/group. (TIF) [file pmed.1002310.s007.tif]

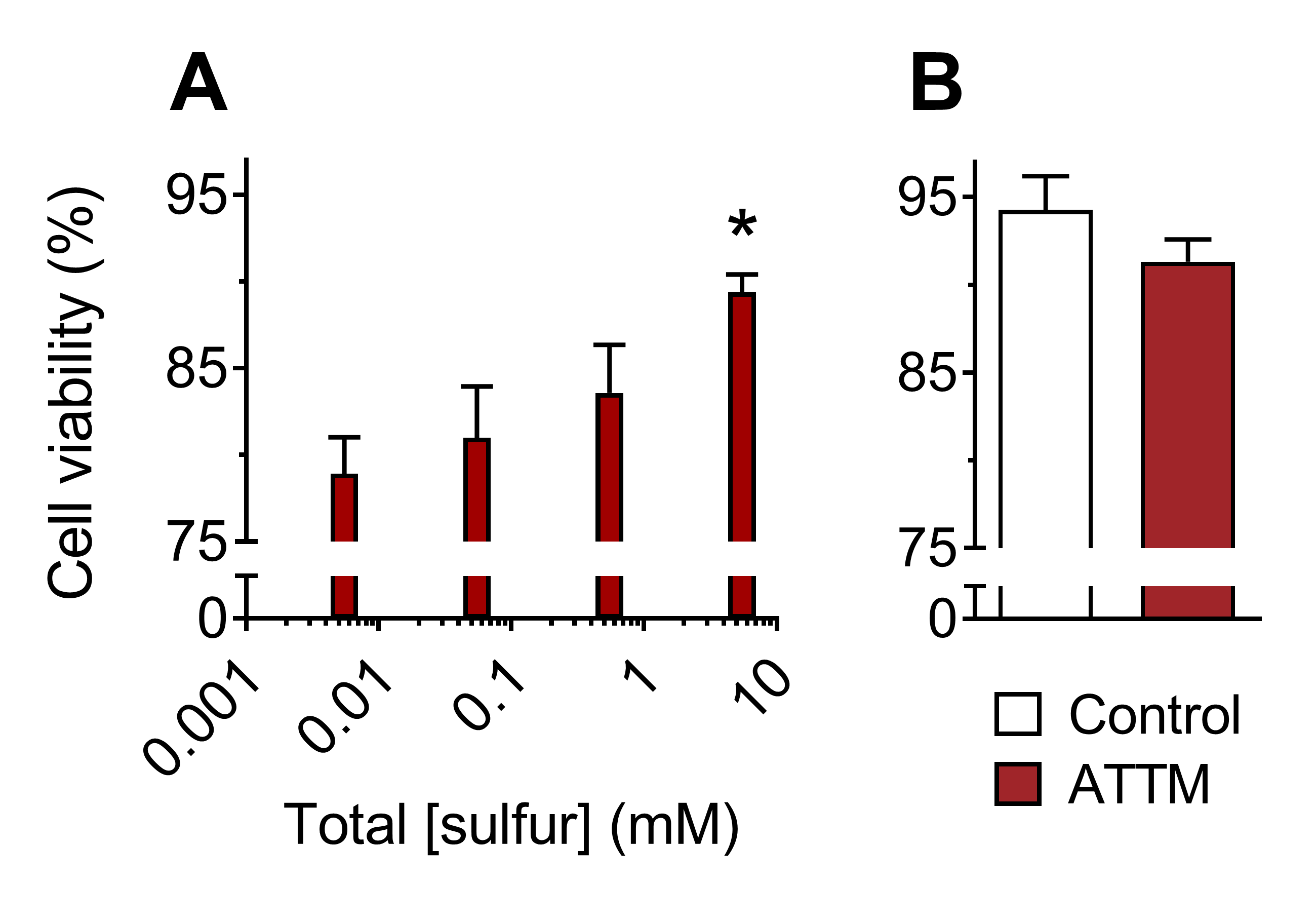

Supplement: S7 Fig — Concentration-dependent improvement in viability following I/R with ATTM treatment (A) and no change in viability in normoxic cells (B). In (B), cells were plated and maintained in a normoxic environment for 24 h, then treated with ATTM (5.5 mM) prior to analysis. *p < 0.05 compared to vehicle (shown in Fig 5), one-way ANOVA plus Dunn’s multiple comparison test. (TIF) [file pmed.1002310.s008.tif]
