# Supplementary material for: Ammonium tetrathiomolybdate following ischemia/reperfusion injury: Chemistry, pharmacology, and impact of a new class of sulfide donor in preclinical injury models
Source: PLoS Med. 2017 Jul 5;14(7):e1002310. doi: 10.1371/journal.pmed.1002310 (PMC5497958; doi:10.1371/journal.pmed.1002310)
Supplement: S1 Text — (DOCX) [file pmed.1002310.s009.docx]

AMMONIUM TETRATHIOMOLYBDATE FOLLOWING ISCHEMIA/REPERFUSION INJURY:

CHEMISTRY, PHARMACOLOGY AND IMPACT OF A NEW CLASS OF SULFIDE DONOR

IN PRECLINICAL INJURY MODELS – SUPPORTING INFORMATION (S1 Text)

**Authors**

Alex Dyson^1,2^, Felipe Dal-Pizzol^1,3^†, Giovanni Sabbatini^1^†, Anna B. Lach^1,2^, Federica Galfo^1,4^, Juliano dos Santos Cardoso^3^, Bruna Pescador Mendonça^3^, Iain Hargreaves^5^, Bernardo Bollen Pinto^1^, Daniel I. Bromage^6^, John F. Martin^1,2^, Kevin P. Moore^7^, Martin Feelisch^8^, Mervyn Singer^1,2^*.

**Affiliations**

^1^Bloomsbury Institute of Intensive Care Medicine, Division of Medicine, University College London, London WC1E 6BT, UK.

^2^Magnus Oxygen Ltd, London WC1R 4AG, UK.

^3^Laboratory of Experimental Pathophysiology, University of Southern Santa Catarina, Criciúma 88806000, Brazil.

^4^Department of Clinical and Experimental Medicine, University of Messina, Messina 98125, Italy

^5^Department of Molecular Neuroscience, Institute of Neurology, University College London, London WC1N 3BG, UK.

^6^Hatter Cardiovascular Institute, University College London, London WC1E 6HX, UK.

^7^Institute for Liver and Digestive Health, University College London, London NW3 2PF, UK.

^8^Clinical & Experimental Sciences, Faculty of Medicine, Southampton General Hospital and Institute for Life Sciences, University of Southampton, Southampton SO16 6YD, UK.

*To whom correspondence should be addressed: Professor Mervyn Singer, m.singer@ucl.ac.uk

†These authors contributed equally to this work

**Short title**: IMPACT OF TETRATHIOMOLYBDATE ON ISCHEMIA/REPERFUSION INJURY

***Monobromobimane (MBB) assay***

Total sulfide (comprising H_2_S, HS^-^ and S^2-^) concentrations under different experimental conditions were measured using a high-performance liquid chromatography (HPLC) based MBB assay. The MBB method relies upon formation of the fluorescent product sulfide dibimane under alkaline conditions. Sulfide dibimane is separated from other reaction products by HPLC and quantified by fluorescence detection. Test samples (100 μl of either NaHS standards, *in vitro* drugs incubated for the desired time period or freshly-centrifuged plasma) were mixed with 100 μl TRIS-HCl (200 mM; adjusted to pH 9.5) and 25 μl MBB (10 mM; dissolved in acetonitrile). After 20 min dark incubation, L-cysteine (10 mM; dissolved in TRIS-HCl) was added to react with the remaining MBB. Following a further 20 min dark incubation, the reaction was stopped by adding 150 μl ice-cold sulfosalicylic acid (200 mM); for plasma samples, this step also precipitates proteins. Samples were then vortexed for 10 seconds, left on ice for 5 min, then centrifuged (1 min at 14000 rcf). The supernatant was stored at 4^o^C in dark conditions until HPLC analysis. Sulfide concentrations were determined using a HPLC system (Waters 474 Scanning Fluorescence Detector; Elstree, UK) and C18 (5 µm) columns (Supelco, Bellefonte, PA). Flow rates of acetonitrile and deionized water were 0.3 and 0.7 ml/min, respectively, and fluorescence detection set to 390 nm for excitation and 475 nm for emission. 10 μl of test sample was injected. AUC was measured using Millennium software (Waters, Elstree, UK) and plotted against a standard curve containing known concentrations of sulfide (NaHS).

***Surgical instrumentation***

Spontaneously breathing animals were anesthetized by 5% isoflurane (Abbott, Maidenhead, UK) in room air (reduced to 2% post-induction) and placed on a heated mat to maintain rectal temperature at 37°C. We use inhaled isoflurane as it allows better cardiorespiratory stability over other agents (in spontaneously-breathing animals). The left common carotid artery and right internal jugular vein were cannulated using 0.96 mm outside diameter PVC tubing catheter (Biocorp Ltd, Huntingdale, Australia). The arterial line was connected to a pressure transducer (Powerlab; AD Instruments, Chalgrove, UK) for continuous monitoring of mean arterial pressure. The venous line was used for subsequent administration of fluids and drugs, as required. A tracheostomy was sited using 2.08 mm external diameter polythene tubing (Portex Ltd, Hythe, UK) to secure and suction the airway. This was connected to a T-piece to maintain anesthesia. In some experiments (PK/PD study and myocardial I/R) the anesthetic was switched to sodium pentobarbitone (Pentoject; Animalcare Ltd, York, UK) through an indwelling intraperitoneal catheter after insertion of vascular lines. The animals were then intubated (blood pressure guided) and ventilated using a small animal physiosuite (Kent Scientific, Torrington, CT). Respiration was volume controlled (10 ml/kg) with a respiratory rate of 80 breaths per minute and a positive end-expiratory pressure of 3 cm H_2_O. These settings ensured adequate post-surgical oxygenation and consistent minute volumes across all animals used. In our hands sodium pentobarbitone allows a more robust respiratory depression as compared to isoflurane, while maintaining global hemodynamics, thus making it more suitable in mechanically ventilated animals.

***Ex vivo metabolic study***

Animals were anesthetized by isoflurane. The right soleus muscle was obtained and immediately transferred to plastic Petri dishes containing ice-cold biopsy preserving solution (isolation medium) containing CaK_2_EGTA (2.77 mM), K_2_EGTA (7.23 mM), Na_2_ATP (5.7 mM), MgCl_2_6H_2_O (6.56 mM), taurine (20 mM), Na_2_ phosphocreatine (15 mM), imidazole (20 mM), dithiothreitol (0.5 mM) and MES monohydrate (50 mM), adjusted to pH 7.4. This method allows storage of tissue with no significant impairment of mitochondrial integrity [1]. The muscle was subsequently dissected with fine forceps and resulting fibers permeabilized with 50 µg saponin in 2 ml isolation medium. This was gently stirred on ice for 20 min. Saponin and metabolites were then removed by washing the fibers three times in ice-cold respiratory medium containing EGTA (0.5 mM), MgCl_2_6H_2_O (3 mM), K-lactobionate (60 mM), taurine (20 mM), KH_2_PO_4_ (10 mM), HEPES (20 mM), sucrose (110 mM) and BSA (1 mg/ml), adjusted to pH 7.4.

Oxygen consumption by the isolated soleus muscle fibers was assessed using a Clark-type oxygen electrode (Rank Brothers, Bottisham, UK), connected to a sealed chamber and maintained at 37^o^C. Oxygen consumption was determined as the fall in oxygen concentration within the chamber over time and subsequently corrected for drift (oxygen consumption by the electrode) and dry weight of each tissue sample. The respiratory medium in the chamber was constantly agitated using a magnetic stirrer. Substrates for mitochondrial respiratory chain complexes I (glutamate, 10 mM; malate, 5 mM) and II (succinate, 5 mM) were added to each chamber by injection through the chamber lid seal. Small bundles (approximately 5 mg) of muscle tissue were then added and the medium oxygenated to approximately 250 μM O_2_ by injecting 3 ml of 100% oxygen gas. Two series of experiments were then performed. In the first series, oxygen consumption was assessed following increasing concentrations of either vehicle (respiratory medium), ATTM (2, 8, 16 and 32 mM; total sulfur) or NaHS (0.5, 1, 2 and 4 mM). Concentrations were increased at 4-minute intervals and oxygen consumption measured in the last minute of each interval. These experiments were performed between 250 and 150 μM O_2_; below this level oxygen consumption becomes dependent on the O_2_ concentration within the chamber. In a second series, tissues respired to hypoxia. Either vehicle, ATTM (2 mM; total sulfur) or NaHS (0.5 mM) was added at 200 μM O_2_. Oxygen consumption was determined at 175, 150, 125, 100 and 75 μM O_2_ and compared to baseline values (recorded just prior to the addition of drugs). In separate experiments, we investigated the effects of non-sulfide containing copper (I and II) chelators neocuproine and cuprizone (1-100 µM).

***Echocardiography***

In some experiments, echocardiography was used to determine myocardial function. Transthoracic echocardiography was performed using a 14 MHz probe scanning at 0–2 cm depth (Vivid 7 Dimension, GE Healthcare, Bedford, UK). Aortic blood flow velocities were determined in the aortic arch using pulsed-wave Doppler. Stroke volume was determined as the product of velocity–time integral (VTI) and vessel cross-sectional area [2]. Heart rate was determined by measuring the time between cardiac cycles. Cardiac output was calculated as the product of stroke volume and heart rate.

***References***

[1] Kuznetsov AV, Veksler V, Gellerich FN, Saks V, Margreiter R, Kunz WS. Analysis of mitochondrial function in situ in permeabilized muscle fibers, tissues and cells. Nat Protoc. 2008; 3:965–976.

[2] Dyson A, Rudiger A, Singer M. Temporal changes in tissue cardiorespiratory function during faecal peritonitis. Intensive Care Med. 2011; 37:1192–1200.
